# Quantitative Profiling of Colorectal Cancer-Associated Bacteria Reveals Associations between *Fusobacterium* spp., Enterotoxigenic *Bacteroides fragilis* (ETBF) and Clinicopathological Features of Colorectal Cancer

**DOI:** 10.1371/journal.pone.0119462

**Published:** 2015-03-09

**Authors:** Katie S. Viljoen, Amirtha Dakshinamurthy, Paul Goldberg, Jonathan M. Blackburn

**Affiliations:** 1 Institute of Infectious Disease & Molecular Medicine, Division of Medical Biochemistry, Faculty of Health Sciences, University of Cape Town, Cape Town, South Africa; 2 Surgical Gastroenterology Unit, Department of Surgery, Groote Schuur Hospital, Cape Town, South Africa; University of Ulster, UNITED KINGDOM

## Abstract

Various studies have presented clinical or *in vitro* evidence linking bacteria to colorectal cancer, but these bacteria have not previously been concurrently quantified by qPCR in a single cohort. We quantify these bacteria (*Fusobacterium spp*., *Streptococcus gallolyticus*, *Enterococcus faecalis*, Enterotoxigenic *Bacteroides fragilis* (ETBF), Enteropathogenic *Escherichia coli* (EPEC), and afaC- or pks-positive *E*. *coli*) in paired tumour and normal tissue samples from 55 colorectal cancer patients. We further investigate the relationship between a) the presence and b) the level of colonisation of each bacterial species with site and stage of disease, age, gender, ethnicity and MSI-status. With the exception of *S*. *gallolyticus*, we detected all bacteria profiled here in both tumour and normal samples at varying frequencies. ETBF (FDR = 0.001 and 0.002 for normal and tumour samples) and *afaC*-positive *E*. *coli* (FDR = 0.03, normal samples) were significantly enriched in the colon compared to the rectum. ETBF (FDR = 0.04 and 0.002 for normal and tumour samples, respectively) and *Fusobacterium* spp. (FDR = 0.03 tumour samples) levels were significantly higher in late stage (III/IV) colorectal cancers. *Fusobacterium* was by far the most common bacteria detected, occurring in 82% and 81% of paired tumour and normal samples. *Fusobacterium* was also the only bacterium that was significantly higher in tumour compared to normal samples (p = 6e-5). We also identified significant associations between high-level colonisation by *Fusobacterium* and MSI-H (FDR = 0.05), age (FDR = 0.03) or *pks*-positive *E*. *coli* (FDR = 0.01). Furthermore, we exclusively identified *atypical* EPEC in our cohort, which has not been previously reported in association with colorectal cancer. By quantifying colorectal cancer-associated bacteria across a single cohort, we uncovered inter- and intra-individual patterns of colonization not previously recognized, as well as important associations with clinicopathological features, especially in the case of *Fusobacterium* and ETBF.

## Introduction

A causal link between specific pathogens and numerous cancers has now been firmly established. Clear evidence exists for example that the vast majority of cervical cancers are directly caused by infection with human papillomavirus (HPV) [1]. Similarly, *Helicobacter pylori* is a known risk factor for the development of gastric cancer and is considered a class I carcinogen by the WHO [2,3].

The possibility of oncogenic bacteria in the colon was already evident in the 1950s when a clinical association between *Streptococcus bovis* bacteraemia/endocarditis and CRC was discovered [4]. Subsequently, multiple studies have demonstrated enrichment with specific bacterial pathogens in faecal or tissue samples of CRC patients, including, *Fusobacterium* spp. [5–7], *S*. *gallolyticus* [8–10], *E*. *faecalis* [11] and Enterotoxigenic *Bacteroides fragilis* (ETBF) [12].

Previously, 16S rRNA profiling of colorectal cancer (CRC) paired tumour and normal biopsies has revealed that while only 3% of biopsy specimens from healthy controls contained any type of bacteria, ~90% of patients with adenomas or carcinomas had 10^3^–10^5^ bacteria in both malignant and macroscopically normal samples [13]. This clearly demonstrates the susceptibility of these patients to colonisation of the normally sterile colonic epithelium––not only in existing tumour tissue, but also in the surrounding macroscopically normal tissue, which may suggest a pre-existing risk to colonisation/infection.

Based on both *in vitro* and *in vivo* observations, bacterially-driven oncogenic mechanisms in CRC have been proposed to include activation of Wnt signaling (ETBF [14], Enteropathogenic *Escherichia coli* (EPEC) [15], *Fusobacterium* [16]), pro-inflammatory signaling (*E*. *faecalis* [17,18], *S*. *gallolyticus* [19,20]) and genotoxicity (EPEC [21], AIEC [22–24]).

The oncogenic potential of these bacteria, as well as suspected bacterial components implicated in the aetiopathogenesis of CRC, are summarized in Table 1.

**Table 1:** Summary of the putative oncogenic mechansims and the bacterial components implicated in CRC pathogenesis for the six bacterial species quantified in this study.

Bacterial species	Support for putative oncogenic mechanism	Suspected bacterial components implicated
Enteropathogenic *Escherichia coli* (EPEC)	Downregulates mismatch repair proteins *in vitro* [21,25]; increases mutational frequency *in vitro* [21].	*espF* [21]
*Escherichia coli* with adherent/and or invasive properties.	Enriched in CRC patients [13,26,27]; CRC-associated strains commonly have genes related to M-cell translocation (*lpfA*) [22]; genotoxicity (*pks*) [22–24,28], or cell cycle modulation (*cnf1*) [23].	*pks* [22–24], *afaC* [22], *lpfA*[22] *cnf1* [23] and *cdt* [23].
*Fusobacterium* spp.	Multiple independent metagenomic studies identify *Fusobacterium* spp. as overrepresented in CRC tissue compared to matched normal mucosa and healthy controls [5–7]. *F*. *nucleatum* increases tumour multiplicity in an APC Min/+ mouse model [29]; Triggers ß-catenin nuclear signaling [16].	*FadA* [16]
Enterotoxigenic *Bacteroides fragilis* (ETBF)	Enriched in faecal samples from CRC patients [12]; Triggers ß-catenin nuclear signaling; induces c-Myc expression and cellular proliferation [14]; increases colitis and tumour in a Min/+ mice model [30].	*B*. *fragilis* toxin (*Bft*)[14]
*Streptococcus gallolyticus*	Enriched in CRC patients with [8–10,31] and without bacteremia [9]. *S*. *infantarius* or its wall extracted antigens promote progression of preneoplastic lesions in rats and promotes pro-inflammatory COX-2 signaling [19,20].	Cell wall extracted antigens
*Enterococcus faecalis*	Enriched in faecal samples from CRC patients [11]; Produces extracellular superoxide [32], promotes inflammation and CRC in IL-10 knockout mice [17,18], and promotes COX-2 related chromosomal instability [33].	Reactive oxygen species (superoxide, hydrogen peroxide)

To date, however, the presence and levels of multiple CRC-associated bacteria have not been examined across a single cohort. Further, to our knowledge, ETBF and *E*. *faecalis* have only been quantified by quantitative PCR (qPCR) in feacal samples of CRC patients, and EPEC has only been quantified in a small CRC cohort with archival FFPE samples [25].

Here we use qPCR to measure the presence of six pathogens, previously reported in association with CRC, in paired adenocarcinoma and adjacent normal mucosal samples; these include *Fusobacterium spp*., *Streptococcus gallolyticus*, *Enterococcus faecalis*, ETBF, EPEC and *afaC*- or *pks*-positive *E*. *coli*.

## Materials and Methods

### Cohort selection

This study consists of two cohorts: Firstly 55 paired colorectal patient samples (adenocarcinoma tissue and adjacent normal mucosa) were collected during surgical resection at the Groote Schuur Hospital, with no pre-selected conditions. Ethical consent was obtained (UCT HREC REF 366/2010) and each patient provided written informed consent to donate samples from the tissues left over after surgical resection to subsequent molecular studies. The second cohort was sourced in order to obtain more patients with sporadic microsatellite instability (MSI). For this cohort, 18 adenocarcinoma samples were selected from archival FFPE specimens that had previously been screened for MSI by immunohistochemistry of the mismatch repair genes *MLH1*, *MSH2* and *MSH6*; these patients were referred for MSI testing because CRC was diagnosed under the age of 50 and/or in two or more first or second degree relatives with an HNPCC-related tumor, regardless of age. For our purpose, we selected patients with MSI (absence of staining of one or more MMR proteins), whom also had mutational screening data available so that we could distinguish between sporadic and HNPCC-based MSI. Of the 18 patients selected two had confirmed mutations in the *MLH1* mismatch repair gene, and were therefore classified as HNPCC. Detailed participant-level characteristics are presented in S1 Table.

### DNA extraction

DNA was isolated from paired patient samples using a Dounce homogenizer and the AllPrep DNA/RNA/Protein kit (Qiagen) according to the manufacturer’s instructions; for the detection of gram-positive bacteria DNA was extracted using the QIAamp DNA Mini Kit (Qiagen). DNA was extracted from ± 25mg of tissue, using the following protocol: each sample was incubated in 180 μl lysozyme @ 20mg/ml for 40 min at 37°C; after adding 20 μl proteinase K, samples were incubated at 56°C until the tissue was completely lysed (at least 4 hours, or overnight if tissue was still visible after 4 hours); samples were next incubated for 30 min in Buffer AL, thereafter, DNA was isolated according to the manufacturer’s instructions. DNA integrity was confirmed in each case by gel analysis (Agilent BioAnalyser 2100; data not shown). For FFPE samples, DNA was extracted from one or two FFPE slides (depending on availability) using the RecoverAll Total Nucleic Acid Isolation Kit (Ambion). After deparraffinization, samples were incubated in 180 μl lysozyme (20mg/ml) for 40 min at 37°C, followed by incubation for 42 hours at 50°C in Proteinase K. For the remainder of the protocol DNA was isolated according to the manufacturer’s instructions.

### MSI testing

For the cohort of fresh-frozen samples, MSI analysis was conducted on DNA extracted from paired tissue samples as well as the corresponding blood samples for each patient, using allelic profiling of the Bethesda panel of microsatellite markers (BAT25, BAT26, D2S123, D5S346, and D17S250), using primers specified by Loukola *et al*. [34]. Samples were classified as microsatellite stable (MSS), microsatellite instable-low (MSI-L) or microsatellite instable-high (MSI-H) if they had 0, 1 or at least 2 of the 5 markers showing instability, respectively [35].

For the FFPE cohort, immunohistochemistry (IHC) had previously been performed for MSH2, MSH6 and MLH1 by the Division of Anatomical Pathology, University of Cape Town. Samples that displayed absence of staining for any of the mismatch repair proteins evaluated were considered MSI-H since IHC has been shown to have high sensitivity (92.7%) and specificity (100%) in detecting MSI [36]. Originally, patients were referred for IHC based on the following criteria: 1) Colorectal cancer diagnosed under the age of 50 years of age and 2) Colorectal cancer diagnosed in two or more first or second degree relatives with an HNPCC-related tumor, regardless of age.

### Primers and control DNA

Primers for the detection of each bacterial species were sourced from the literature or designed in-house, and their specificity was confirmed using Primer BLAST [37]. All primers, along with their limits of detection (LODs) and qPCR efficiencies, are listed in S2 Table.

The following reagents were obtained through the NIH Biodefense and Emerging Infections Research Resources Repository, (NIAID, NIH) as part of the Human Microbiome Project: *Streptococcus gallolyticus* subsp. *gallolyticus*, Strain TX20005, HM-272D; Genomic DNA from *Bacteroides fragilis*, Strain 3_1_12, HM-20D, Genomic DNA from *Clostridium difficile*, Strain NAP07 (CDC#2007054), HM-88D; Genomic DNA from *Enterococcus faecalis*, Strain HH22, HM-200D; Genomic DNA from *Escherichia coli*, Strain B171, NR-9297; and Genomic DNA from *Fusobacterium nucleatum* subsp. *polymorphum*, Strain F0401. ETBF genomic DNA (ATCC43858) was kindly provided by Dr Annalisa Pantosti from the Istituto Superiore di Sanità, Italy. DNA from enterohemorrhagic *E*. *coli* (to confirm EPEC identity) was kindly supplied by Dr. Anthony Smith at the National Institute for Communicable Diseases, South Africa. DNA from AIEC (strains HM358, HM229 and HM334) was kindly provided by Dr. Barry Campbell from the University of Liverpool, UK.

### qPCR amplification conditions

Experiments were performed in triplicate on a Roche LightCycler 480 Real-Time PCR System in 96-well format, using 50 ng patient DNA per well. Separate assays were performed for each bacterial gene detected; the cycling conditions are specified in S3 Table. EPEC (*eaeA*, *bfpA* and *stx1* and *stx2*), ETBF and *S*. *gallolyticus*, were each detected in 20 μl reactions using SensiFAST SYBR No-ROX Kit (Bioline); AIEC, Fusobacterium spp. and *E*. *faecalis* were each detected in 25 μl reactions using Maxima SYBR green qPCR Master Mix (Thermo Scientific). In order to increase specificity, it was necessary in some cases to perform touchdown PCR, whereby the annealing temperature is lowered in a stepwise manner to discourage amplification of off-targets during the first 10 cycles of PCR [38]; Touchdown qPCR was performed for detection of EPEC (*bfpA* and *eaeA*), *S*. *gallolyticus*, ETBF, EHEC (*stx1* and *stx2*) and AIEC (*afaC*).

### qPCR quantification

For each qPCR assay, absolute quantification was performed using a standard curve, which was constructed using serially diluted genomic DNA from the relevant positive control strain. The concentration of bacterial DNA found was expressed in terms of genome copies by calculating the weight of one genome copy for each species as used by Dolezel *et al*.[39]: DNA content (pg) = genome size (bp)/(0.978 x 10^9^).

For example, *Fusobacterium* spp. have an estimated genome size of 2.2 Mb and since one molecule of double stranded DNA of length 978 Mb weighs approximately one picogram, a single *Fusobacterium* spp. genome weighs approximately 2.25 fg (2.2Mb/978Mb = 0.00225 pg) and therefore 1 ng of DNA from *Fusobacterium* spp. equates to 444,545 copies (1000pg/(2.2Mb/978Mb)) of the bacterium (Table 2).

**Table 2:** Estimates of bacterial genome copies per nanogram of bacterial DNA.

	Strain, genome size	Number of target gene copies/genome assumed.	Estimated bacterial copies/ng bacterial DNA.
EPEC (*eaeA/bfp*)	E2348/69, 4.97 Mb	1	2 x 10^5^
ETBF (*bft*)	3_1_12, 5.49 Mb	1	1.8 x 10^5^
*E*. *faecalis* (16s rRNA)	V583, 3.34 Mb	4[40]	1.2 x 10^6^
*Fusobacterium* spp. (16s rRNA)	NA, 2.8 Mb[41]	5[41]	1.8 x 10^6^
AIEC (*afaC*)	LF82, 4.88 Mb[42]	1	2 x 10^5^
AIEC (*ClB*)	LF82, 4.88 Mb	1	2 x 10^5^
*S*. *gallolyticus* (*sodA*)	UCN34, 2.35[43]	1	4 x 10^5^
EHEC (*stx1*)	O157:H7, 5.6	1	1.8 x 10^5^
EHEC (*stx2*)	O157:H7, 5.6	1	1.8 x 10^5^

In the case of AIEC strains, genome size may vary substantially between strains, since these strains are classified according to phenotypic traits and not sequence similarity. We opted to use the prototypical LF82 AIEC strain, which has a genome size of 4.88 for quantification.

In all cases, data were normalised to total genomic DNA and represented as number of bacterial genomes per 50 ng human DNA, thereby effectively normalising the qPCR data to tissue size.

Positive control standards were spiked with the same amount of human genomic DNA (extracted from uninfected human cell cultures) as used in the patient sample reactions (50ng). The limit of detection (LOD) was defined as the lowest concentration at which a positive result (correct meltcurve) could be obtained in at least 50% of replicates (see S2 Table for details). For all assays except *ClB* and *afaC* at least 70% of replicates were positive at the relevant LOD. In cases where results were inconsistent (1/3 replicates positive), samples were retested and taken as positive if a positive meltcurve was obtained in both runs (the results were then averaged across the two runs to obtain quantitative data).

### qPCR quantification in FFPE samples

We first evaluated the quality of DNA extracted from archival FFPE slides (which had been stored between 2 and 23 years) using three primer pairs designed to amplify 100bp, 200bp and 300bp amplicons of the *GAPDH* gene [44]. For most samples we detected either a very faint or no visible band at 100bp; whilst a 200bp amplicon could only be amplified in a few samples. On testing a shorter amplicon (69bp) of the *COX1* gene (which we found to be stably expressed in our cohort and is therefore assumed to have no significant differences in copy number between samples), all samples could be amplified by qPCR; the difference in cycle threshold (Ct) between the highest and lowest quality sample was 9.3. We therefore redesigned the reverse primers for bacterial detection to shorten the resulting amplicons to 60–70bp, and used the *cox1* results to account for degradation in our bacterial quantification. In the case of *eaeA*, two reverse primers were designed, one that detects intimin subtypes epsilon, gamma, zeta, alpha, pi, rho, beta, lambda, iota, kappa, eta, delta, xi, mu, kappa and jota; while the second was designed to specifically detect intimin theta (which was found in both EPEC-positive MSI-H samples from the fresh-frozen cohort). The efficiencies for the *COX1* qPCR was calculated using 5-fold serial dilutions constructed using for a high- and low quality patient sample, as 1.96 and 2, respectively. A ‘fold change’ value was then calculated for *COX1* in each sample, using the ΔΔCt method and the mean Ct across 6 randomly selected DNA samples from the fresh-frozen (high-quality DNA) sample cohort was used as reference (the maximum ΔCt between fresh-frozen samples was 1.8). These sample-specific ‘fold change’ values for *COX1* between FFPE samples to be tested and the reference set of fresh-frozen samples were used as a correction factor to adjust for DNA sample quality. A theoretical limit of detection was also calculated for each sample by multiplying the correction factor for each sample with the LOD that had previously determined for high quality DNA. After performing absolute quantification, the result was multiplied by the correction factor for each sample. The validity of this method was assessed by comparing *Fusobacterium* quantitation obtained from DNA extracted from fresh frozen samples to that of the matched FFPE samples (which we had available for four patients); after removing a single outlier sample, the Pearson’s correlation coefficient was 0.94, and the median fold change between matched fresh-frozen and FFPE samples was 1. FFPE samples that tested negative for *Fusobacterium* were set to ‘NA’ for downstream analysis, since the negative results could be due either to sample quality or to absence of the bacterium.

### Statistical analyses

In order to assess quantitative differences between paired tumour and normal samples for each bacterium, we used the Wilcoxon signed rank test applied to the subset of samples, which had at least one positive sample in a pair (tumour or normal).

**Fig 1:**
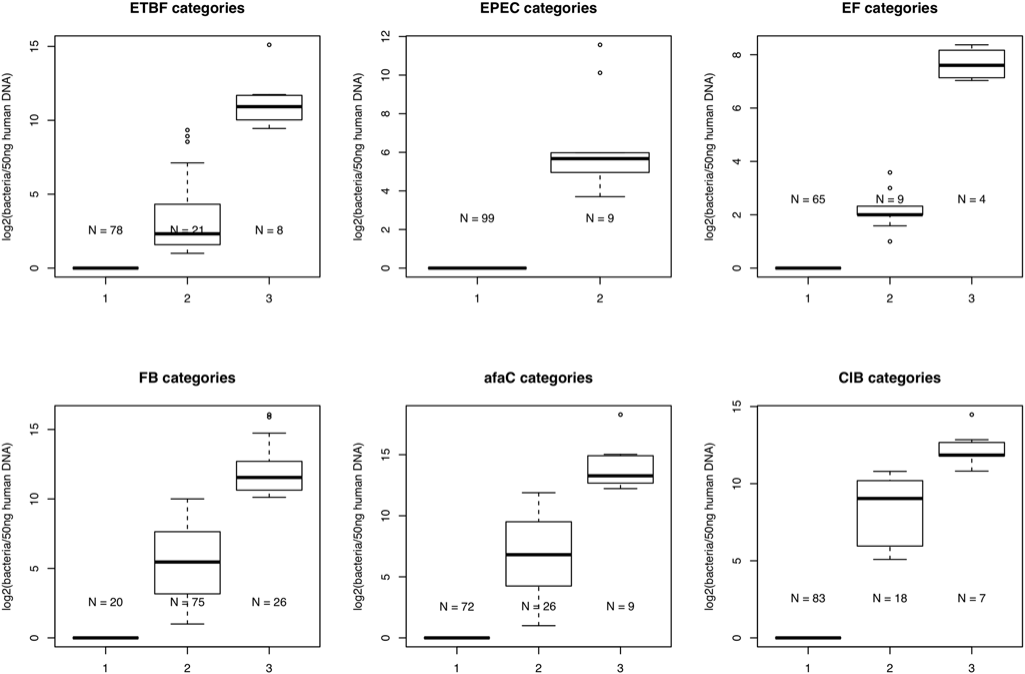
Levels of colonization by each bacterium/gene were categorized using the third quartile (taken across colonisation-positive samples) as a cutoff for high- or low-level colonisation. Categories: 1 (No colonisation), 2 (low colonisation), 3 (high colonisation). In the case of EPEC, because there were so few EPEC-positive patients (N = 6), samples were analysed as positive or negative only. EF: *E*. *faecalis*; FB: *Fusobacterium*.

To assess the association between each bacterium and clinicopathological features, we compared a) samples with vs. without colonisation by a particular bacterium and b) samples with high vs. low/no-colonisation by a particular bacterium. Except for *Fusobacterium*, all other bacterial quantitative data suffered from zero-inflation due to the large number of colonisation-negative patients, which lead to unequal variances between groups. We therefore converted the quantitative data to categorical data where for each bacterium, samples were categorised as ‘no-colonisation’, ‘low-colonisation’ or ‘high-colonisation’. Quantitative data (copies/50ng) were log2 transformed and samples with no-colonisation were arbitrarily set to 1 before log2 transformation; the third quartile (calculated across colonisation-positive cases only) was used to discriminate low- and high-colonisation cases (see Fig. 1 for categories). Associations with clinicopathological features were examined using Fisher’s exact test. Meanwhile, in the case of *Fusobacterium* (where the data was normally distributed), we used the Kruskal-Wallis test to evaluate differences between groups stratified by the clinicopathological parameter of interest. Results with an FDR ≤ 0.05 after applying multiple-testing-correction (Benjamini-Hochberg method) over all clinicopathological comparisons made, for each species, were considered significant.

## Results

### Clinicopathological characterization

The clinicopathological characteristics of the patients in cohort 1 (fresh-frozen samples) are summarized in Table 3. Briefly, the mean age of patients was 59 (SD±15.3), while gender was divided equally. The samples for which MSI-testing was performed (N = 23) included 7 MSI-H (including 4 HNPCC patients) and 3 MSI-L patients. The majority of cases were stage II or III cancers (81.6%), while stage I and IV cancers accounted for 12.2% and 6.1% of cases, respectively. The cohort consists of 60% rectal and 40% colon cancers, with proximal cancers accounting for 45% of the colon cancers. Further, the majority of our cohort is mixed-ancestry (70.4%), while patients of caucasian (14.8%), black (11.1%) and indian ethnicities (3.7%) made up the rest of the cohort.

**Table 3:** Clinicopathological characteristics of the cohort of fresh-frozen tissues (N = 55).

Feature (patients with missing data)	Number of patients N = 55
Mean age (2)	59 (SD±15.3)
BMI (4)	26.8 (SD±4.7)
Gender (1)	
*Female*	27 (50%)
*Male*	27 (50%)
MSI status (23)	
*MSS*	22 (68.8%)
*MSI-H*	7 (21.9%)
*MSI-L*	3 (9.4%)
CRC Type	
*HNPCC*	6 (10.9%)
*Sporadic*	49 (89.1%)
Tumour stage (6)	
*I*	6 (12.2%)
*II*	18 (36.7%)
*III*	22 (44.9%)
*IV*	3 (6.1%)
Tumour site (5)	
*Ceacum*	4 (8%)
*Ascending colon*	1 (2%)
*Hepatic flexure*	1 (2%)
*Transverse colon*	3 (6%)
*Splenic flexure*	1 (2%)
*Descending colon*	3 (6%)
*Sigmoid colon*	3 (6%)
*Rectosigmoid junction (RSJ)*	4 (8%)
*Rectum*	30 (60%)
Radiation/Chemo received before resection (2)	
*Yes*	22 (41.5%)
*No*	31 (58.5%)
Ethnicity (1)	
*Black*	6 (11.1%)
*Caucasian*	8 (14.8%)
*Indian*	2 (3.7%)
*Mixed-Ancestry*	38 (70.4%)

In the case of age and BMI mean values and their standard deviations (SD) are reported. The numbers in column 1 in brackets represent the number of patients with missing data in each category.

Given the association between EPEC and MSI demonstrated by Maddocks et al.[25] and here, as well as the reported association between the levels of colonization by *Fusobacterium* spp. and MSI status, since we had limited numbers of MSI-H samples in the original fresh-frozen cohort, we sourced additional MSI-H samples in order to increase statistical power to measure the relationship between *Fusobacterium* or EPEC and MSI status. To do this, we leveraged archival FFPE samples from Groote Schuur Hospital, Cape Town, South Africa, for which immunohistochemistry of the mismatch repair genes MLH1, MSH2 and MSH6 had been conducted previously.

### Bacterial quantification

We quantitated CRC-associated bacteria in adenocarcinoma and matched normal mucosal samples by qPCR, using a serial dilution of genomic DNA from each bacterium as standards, and found varying levels of colonisation in tumour and/or adjacent normal mucosa for all bacteria measured, except *S*. *gallolyticus*, for which no positive samples were found. While the association between *S*. *gallolyticus* bacteremia or infective endocarditis and CRC is well established [10], we found only one study, by Abdulamir *et al*. [9], where *S*. *gallolyticus* was measured in CRC patients without a history of bacteremia or infective endocarditis. That study found that 4% of healthy controls but 48.7% and 32.7% of CRC patients with or without bacteremia were infected with *S*. *gallolyticus* in the relevant colonic tissue [9]. In contrast, we did not detect *S*. *gallolyticus* in any of our adenocarcinoma or matched normal mucosa samples using the same primers used by Abdulamir *et al*. [9] for conventional PCR and qPCR. We note that the levels reported in that study were typically very low and we also note that none of our cohort had any reported history of bacteremia/bacterial-endocarditis. It is important in this regard that our qPCR assay was very sensitive (LOD = 5 copies/50ng DNA) and allowed for the detection of gram-positive bacteria such as *S*. *gallolyticus* by the addition of lysozyme to the homogenized human tissue prior to DNA extraction; this suggests that the discrepancy between our results and those of Abdulamir *et al*. might be explained by a) differences in sample preparation, b) ethnic differences in the susceptibility to colonization by *S*. *gallolyticus* or c) geographical differences in *S*. *gallolyticus* strains found in Southern Africa that may have precluded detection of the bacterium in our cohort. Further investigation is therefore required to clarify this discrepancy.

**Fig 2:**
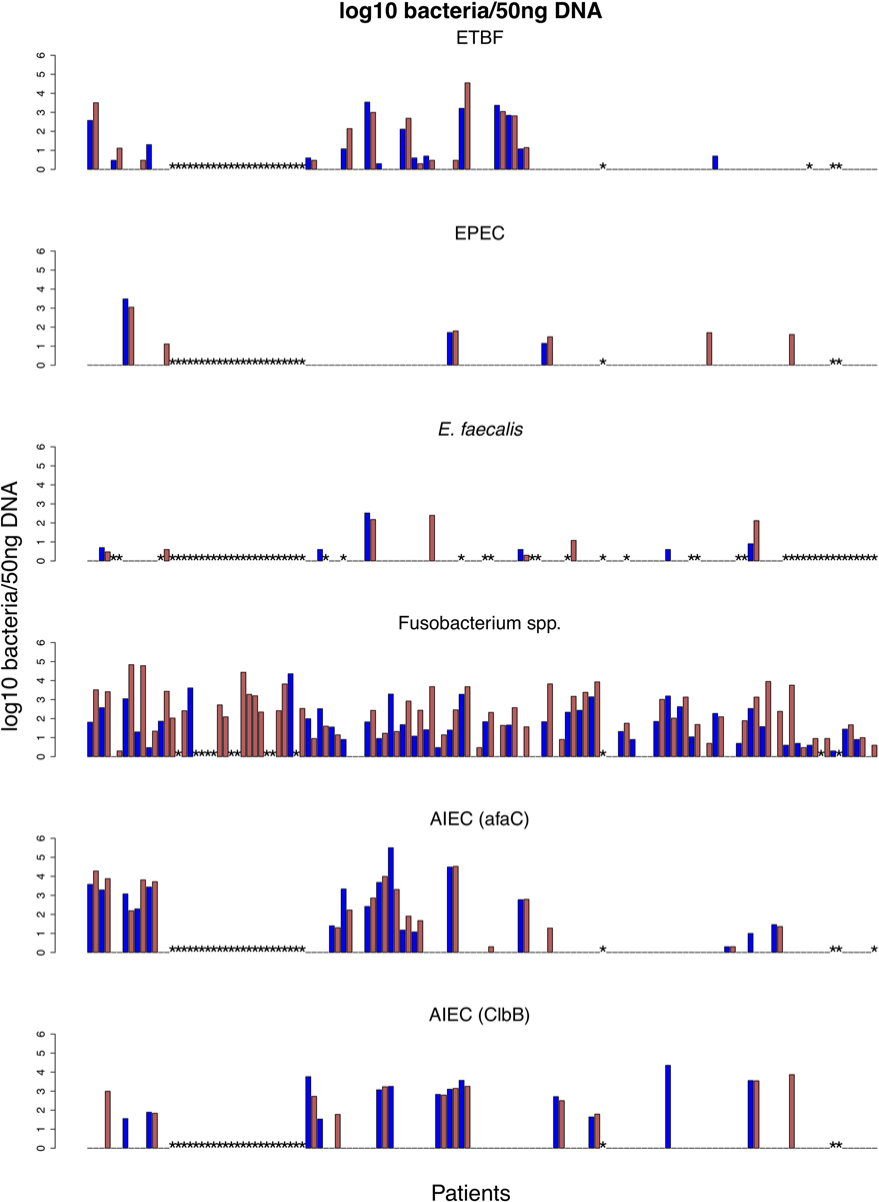
qPCR quantification of bacteria in paired patient samples, expressed as log10 bacteria/50ng of patient DNA. Each bar represents one samples (either tumour or normal) and the order of samples are the same for each bacterium. Red (tumour); blue (normal); *(Not determined).

Of the bacteria that we detected, *Fusobacterium* was by far the most common, occurring in 82% and 81% of paired tumour and normal samples, respectively, with 80% concurrent colonisation in paired samples. *Fusobacterium* was also the only bacterium that was significantly higher in tumour compared to normal samples (p = 6e-5, Wilcoxon signed rank test), which is in agreement with previous studies [5,7,45]. The qPCR results are summarized in Fig. 2 and Table 4.

**Table 4:** Quantification of bacteria in colorectal cancer and adjacent normal tissues.

Pathogen	Colonisation rate T (%)	Colonisation rate N (%)	Concurrent colonisation in T & N (%)
*Fusobacterium*	58/71 (82%)	48/59 (81%)	43/54 (80%)
AIEC (*afaC*)	19/53 (36%)	17/54 (31%)	16/20 (80%)
AIEC (*pks*)	12/54 (22%)	13/55 (24%)	9/16 (56%)
*E*. *faecalis*	11/40 (28%)	7/38 (18%)	5/10 (50%)
ETBF	14/54 (26%)	15/53 (28%)	12/17 (71%)
EPEC	6/54 (11%)	3/54 (6%)	3/6 (50%)
*S*. *gallolyticus*	0/45 (0%)	0/45 (0%)	0/45 (0%)

T and N denote adenocarcinoma and adjacent normal mucosa, respectively. Rates of concurrent colonisation in T and N samples were calculated as a fraction of the number of patients who were infected in T and/or N with a particular bacterium.

We sequenced *Fusobacterium* amplicons from 10 of the samples in our cohort to confirm melt curve matches, but could not in all cases determine strain-level identity from these amplicons (data not shown). *F*. *nucleatum* is the only *Fusobacterium* species that has been associated with CRC in the literature to date so it seems reasonable to suppose that in our study the *Fusobacterium* spp is *F*. *nucleatum*. However, to confirm this would have required a metagenomic sequencing approach to explore *Fusobacterium* spp representation in more detail, since it is not yet known which other *Fusobacterium* spp. might be relevant to the disease; this was beyond the scope of the present study so we refer hereafter exclusively to *Fusobacterium* spp.

In our cohort, we detected ETBF in 14/54 (26%) of colorectal adenocarcinomas and 15/53 (28%) of adjacent normal mucosa samples and 71% of ETBF+ patients were infected in both adenocarcinoma and matched adjacent normal samples. This is consistent with previous studies on faecal samples, which have reported ETBF in ±12% of healthy controls [12,46], 27% of patients with diarrhea [46], and 38% of patients with CRC [12] with colonisation rates appearing to vary widely by geographical location [30].

Although Balamurugan *et al*. demonstrated significantly higher levels of faecal *E*. *faecalis* in CRC patients compared to healthy controls [11], to our knowledge, ours is the first study to quantitatively measure *E*. *faecalis* in paired adenocarcinoma (28% *E*. *faecalis-*positive) and normal mucosa samples (18% *E*. *faecalis-*positive) with 50% of infected patients being infected in both adenocarcinoma and matched normal mucosa samples. We did not however find any significant clinical associations with *E*. *faecalis* colonisation.

To investigate the presence of *E*. *coli* genes that are commonly found in AIEC and which might be relevant to oncogenesis, we quantified the presence of *ClB* (part of the *pks* genomic island) and *afaC* (present in all operons of the afimbrial adhesin family) in paired CRC samples; *pks+ E*. *coli* has previously been detected in 55–67% of CRC patients [22,47], compared to 8% of healthy controls [22]. By contrast, in our cohort, 22% of adenocarcinomas and 24% of adjacent normal mucosa samples were *pks*+, and 56% of *pks+* patients were infected in both adenocarcinoma and matched normal mucosa samples. We also detected *afaC* in 36% and 31% of adenocarcinoma and normal mucosa samples, respectively, and found that 80% of *afaC+* patients were infected in both adenocarcinoma and matched normal mucosa samples; this is much lower than that found by Prorok-Hamon *et al*., who found 67% of CRC patients to be *afaC+* compared to 17% of controls [22]. This discrepancy could be explained by our relatively high LOD for *afaC* and *pks*. Lastly, in contrast to Buc *et al*. [47], who found *pks* to be more common in distal compared to the proximal colon, we did not find a significant association between the presence of *pks* and site of disease. It should be noted that while we examined the presence of *pks* and *afaC*, many other AIEC-related genes that might be relevant to oncogenesis exist, including cyclomodulins [47] and *lpfA* [22].

### ETBF and afaC-positive *E*. *coli* are significantly enriched in the colon compared to the rectum of CRC patients.

**Fig 3:**
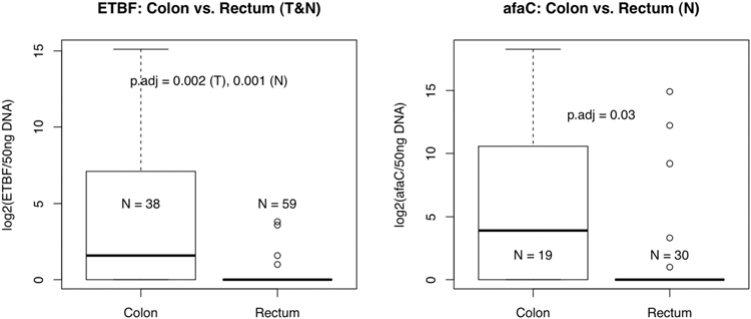
ETBF and afaC+ *E*. *coli* are significantly more prevalent in colon vs. rectal cancers. This applies to both tumour and normal tissue in the case of ETBF (FDR = 0.002, 0.001, respectively) and normal tissue only in the case of afaC (FDR = 0.03).

As shown in Fig. 3, the presence of ETBF and *afaC*-positive strains were significantly associated with the colon compared to the rectum in normal samples (FDR = 0.001 and 0.03, respectively), as well as in tumour samples in the case of ETBF (FDR = 0.002). We did not find any significant differences in colonisation between the proximal and distal colon for any of the bacteria in this study.

### Colonisation by ETBF and high-level colonisation by *Fusobacterium* are associated with late-stage CRC.

**Fig 4:**
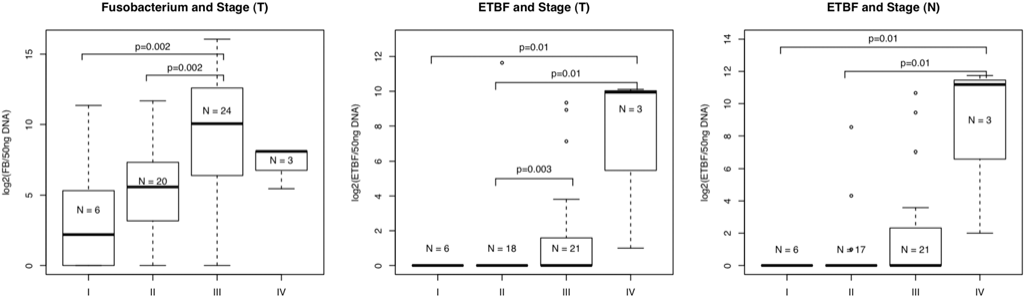
ETBF and *Fusobacterium* are found at significantly higher levels in late stage (III/IV) cancers. For *Fusobacterium*, individual stages were compared in a pairwise manner using Dunn’s test. For ETBF, individual stages were compared in a pairwise manner using Fisher’s exact test. *Fusobacterium* is found at significantly higher levels in stage III CRCs compared to stage I or II CRCs. ETBF is found more frequently in stage III or IV CRCs compared to stage I or II CRCs; and in the corresponding normal mucosa of stage IV CRCs compared to stage I CRCs.

As shown in Fig. 4, the presence of ETBF was significantly associated with stage of disease (Fisher’s exact, FDR = 0.04 and 0.002 for normal and tumour samples, respectively). Similarly, in the case of *Fusobacterium*, late stage (III/IV) tumour samples were significantly associated with high-level colonisation by *Fusobacterium* (Kruskal-Wallis, FDR = 0.03).

In order to investigate which CRC stages were significantly different in terms of the level of colonisation by *Fusobacterium* we used Dunn’s test to compare individual stages in a pairwise manner. *Fusobacterium* levels were significantly higher in stage III CRCs compared to stage I or II CRCs (p = 0.002 for both comparisons). For ETBF, for which we found a difference in the presence or absence of ETBF between stages, individual stages were compared in a pairwise manner using Fisher’s exact test. ETBF was found more frequently in stage III or IV CRCs compared to stage I or II CRCs (stage I vs. IV p = 0.01; stage II vs. stage IV p = 0.01; stage II vs. stage III p = 0.003) as well as in the normal mucosa of stage IV CRCs compared to stage I CRCs (stage I vs. IV p = 0.01; stage II vs. IV p = 0.01).

**Fig 5:**
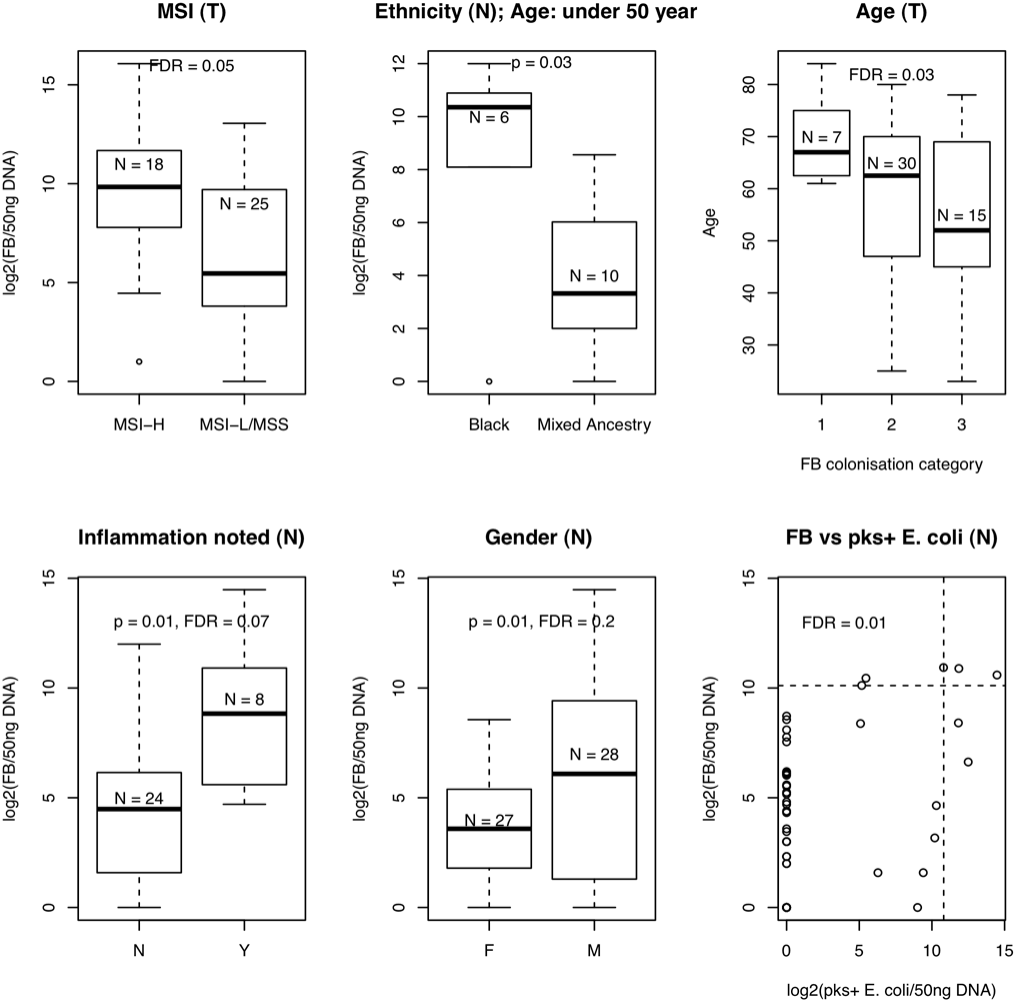
*Fusobacterium* clinicopathological associations. High-level colonisation by *Fusobacterium* is significantly more prevalent in younger patients, males and patients of Black ethnicity. Due to the disproportionately high number of young, black patients seen in our cohort the relationship between ethnicity and levels of colonisation by *Fusobacterium* was assessed using the subset of patients ≤ 50 years. A borderline significant relationship was seen between high-level colonisation by *Fusobacterium* and MSI-H compared to MSS/MSI-L (In our cohort three MSI-L cases were included with the MSS cohort). The vertical and horizontal dotted lines in the bottom right Fig. represent the cutoff for high-level colonisation by *pks+ E*. *coli* and *Fusobacterium*, respectively (see methods for further detail). FB: *Fusobacterium*; B: Black; C: Caucasian; I: Indian; M: Mixed Ancestry; N: normal tissue; F: Female; M: Male.

High-level colonisation by *Fusobacterium* also seems to correlate with chronic inflammation in CRC. For example, McCoy *et al*. found a significant positive correlation between *Fusobacterium* species abundance and local inflammation in adenoma cases [6] whilst we found that there is a trend towards high-level colonisation by *Fusobacterium* in patients with noted inflammation in normal tissue (Kruskal-Wallis test, p = 0.01, FDR = 0.07) (Fig. 5) and tumour tissue (Kruskal-Wallis, p = 0.18, FDR = 0.2). We also found a positive association between high levels of colonisation by *Fusobacterium* and *pks*-positve *E*. *coli* in normal tissue (Fisher’s exact, FDR = 0.007) or EPEC in tumour tissue (Fisher’s exact, p = 0.08, FDR = 0.2). These data suggest that certain individuals may be more susceptible to bacterial colonisation and inflammation of the normally sterile colonic epithelium.

### Further clinical associations with high-level colonisation by *Fusobacterium*

We found a significant relationship between high-level colonisation by *Fusobacterium* and MSI-H, compared to samples that were MSS or MSI-L (Kruskal-Wallis, FDR = 0.05), Fig. 5. We also found a significant increase in *Fusobacterium* levels in CRCs of younger patients (< 60 years), (Kruskal-Wallis, FDR = 0.03) with 31% vs. 11% of patients under or over the age of 60 falling into the *Fusobacterium*-high group of colonisation (Fig. 5). In normal samples, we also noted a trend towards high-level colonisation in males compared to females (Kruskal-Wallis, p = 0.05), but this was not significant after multiple testing correction, Fig. 5.

In order to objectively assess the levels of colonisation by *Fusobacterium* between different ethnic groups we used subsets of the data to account for the significant difference in patient age by ethnicity (ANOVA p = 7.2e-6); across all patients, the mean age of black patients was 36, that of mixed ancestry patients was 58, and that of white patients was 77. We therefore performed two age- and gender-matched comparisons: a) black patients (mean age = 35, N = 6) vs. mixed ancestry patients under the age of 50 (mean age = 42, N = 10) and b) caucasian patients (mean age = 77, N = 8) vs. mixed ancestry patients over the age of 70 (mean age = 72, N = 19). *Fusobacterium* was found at significantly higher levels in black patients compared to their age-matched mixed ancestry counterparts in adjacent normal samples, (Kruskal-Wallis, p = 0.03, Fig. 5), but not in tumour samples (Kruskal-Wallis, p = 0.6). No significant differences were found between age-matched caucasian and mixed ancestry patients in terms of *Fusobacterium* colonisation levels.

Finally, the *Fusobacterium*-high group was also significantly associated with the presence of *pks-*positive *E*. *coli* in normal samples (Fisher’s exact, FDR = 0.01) and two of the three EPEC+ tumours were also infected with *Fusobacterium*-high (Fisher’s exact, p = 0.08, FDR = 0.2), Fig. 5.

### EPEC detection and characterisation

In the fresh-frozen cohort, we detected EPEC via the intimin gene (*eaeA*) in 11% and 6% of tumour and normal samples, respectively; with 50% concurrent colonisation in paired samples. Colonisation levels varied from ±13–3037 bacteria/50ng of DNA extracted. Notably, all EPEC-positive cases (N = 6) were identified as atypical EPEC (aEPEC) by screening for *bfpA* (present in typical EPEC) and *stx1* (present in shiga-toxin producing *E*. *coli* (STEC)), neither of which were present in any of our samples. aEPEC has not been previously reported in association with CRC, although the EPEC detected in FFPE CRC samples by Maddocks *et al*. [25] could be aEPEC since they only profiled *eaeA*, and not *bfpA* or *stx1*.

No significant clinical associations were found for EPEC––this is not surprising given the small number of EPEC positive patients (N = 6). However, of the six patients infected with EPEC, 67% (2/3) of sporadic MSI-H cases (fresh-frozen cohort), and only 9% (2/22) of MSS were EPEC-positive; the remaining two EPEC-infected patients were of unknown MSI status. Therefore, similar to *Fusobacterium*, there seems to be a trend towards colonisation by EPEC in sporadic MSI-H patients. Furthermore, in light of the effect of intimin subtype (of which there are currently 27 known variants [48]) on tissue tropism [49–51] we sequenced the 150bp amplicon amplified during intimin detection, which is located in the variable region of intimin and identified intimin theta exclusively in the two EPEC-positive MSI-H cases and in one case with unknown MSI-status. In the remaining EPEC-positive samples, intimin subtype could not be conclusively identified based on the 150bp product, but produced equal BLAST scores for the intimin subtypes: zeta 2&3, alpha 2, pi, iota 1, delta, beta 2, epsilon 2&8, jota and lambda in all of the remaining samples. In samples with concurrent colonisation in paired samples, the 150bp product sequences were identical. Intimin sequencing results are summarized in Table 5.

**Table 5:** Summary of BLAST search query to identify intimin subtypes.

Sample	Highest scoring BLAST hits	% identity	Patient MSI status (T)	*MLH1* hypermethylated	Paired T&N sequences identical
**44T**	theta	100	MSI-H	Y	Y
**44N**	theta	99	MSI-H	N	Y
**63T**	theta	100	MSI-H	Y	NA
**34N**	zeta 2&3, alpha 2, pi, iota 1, delta, beta 2, epsilon 2&8, jota, lambda	100	MSS	N	Y
**34T**	zeta 2&3, alpha 2, pi, iota 1, delta, beta 2, epsilon 2&8, jota, lambda	100	MSS	N	Y
22T	theta, gamma	98	ND	ND	NA
45T	zeta 2&3, alpha 2, pi, iota 1, delta, beta 2, epsilon 2& 8, jota, lambda	97	ND	ND	NA
29N	zeta, alpha 2, pi, iota 1, delta, beta 2, epsilon, jota, lambda	100	MSS	ND	Y
29T	zeta 2&3, alpha 2, pi, iota 1, delta, beta 2, epsilon2& 8, jota, lambda	100	MSS	ND	Y

Samples highlighted in bold were used to determine the effect of FFPE fixation on the ability to detect EPEC by qPCR. ND: not determined.

Our finding that intimin theta was exclusively identified in MSI-H EPEC positive cases (both located in the caecum), and in one case of unknown MSI status (located in the rectum) is interesting. Moreover, the two MSI-H patients infected with intimin-theta aEPEC were also the only two patients (with available MSI data) where *MLH1* was hypermethylated, as determined by methylation-specific qPCR. Both these patients were also infected with high levels of *Fusobacterium* (2730 and 68700 copies/50ng in tumour samples). Our data contrast with the work of Maddocks *et al*. who recently demonstrated *in vitro* EPEC-induced depletion of the mismatch repair proteins occurring at the protein level, despite an apparent increase in *MLH1* and *MSH2* mRNA following infection of HT29 cells with EPEC (strain E2348/69) [21]. Maddocks *et al*. concluded that EPEC-induced depletion of MLH1 and MSH2 proteins was dependent on mitochondrial targeting of the EPEC effector protein EspF and that this depletion significantly increased the mutational frequency of infected cells [21]. Further work therefore seems needed to reconcile the apparently differing molecular origins of MLH1 protein depletion suggested by the cell-line-based studies of Maddocks *et al*. and our studies on clinical samples.

Lastly, although we did not sequence the entire intimin gene, the 150bp amplified sequences were consistently identical within but not between patients, suggesting that strains isolated from tumour or normal biopsies from a given patient are identical, in agreement with the findings by Martin *et al*. concerning *E*. *coli* strains in paired CRC samples [26].

Next, given the reported relationship between EPEC and MSI *in vitro* [21,25] as well as the relationship between intimin theta+ aEPEC and MSI seen here, we sourced 18 additional MSI-H samples from archival FFPE samples. However, none of which tested positive for EPEC, but because the median level of EPEC colonisation across EPEC-positive samples from the fresh-frozen cohort was relatively low (51 copies/50ng DNA), we investigated whether the level of degradation in the FFPE samples precluded detection in these samples. To this extent we compared the qPCR results from fresh-frozen (150bp amplicon) and matched archival FFPE samples (70 bp amplicon) for three EPEC-positive patients (5 EPEC-positive T or N samples). EPEC could only be detected in one of the five matched FFPE samples––the sample that displayed the highest level of colonisation (3037 copies/50ng) in the fresh frozen tissue. Further, the median estimated LOD for the FFPE samples was 191 copies/50ng DNA (see Methods for further details), which is higher than the median level detected in fresh frozen samples (51 copies/50ng DNA). We therefore conclude that if EPEC were present in the MSI-H FFPE samples at levels similar to that seen in fresh-frozen samples, the level of degradation in the FFPE samples would have precluded detection of EPEC, even when attempting to amplify a 70bp amplicon.

## Discussion

There are an increasing number of reports in the literature of specific bacteria enriched in CRC patients compared to healthy controls. Here, our goal was to simultaneously characterise these bacteria across a single cohort in both tumour and histologically normal (as identified by a qualified anatomical pathologist) adjacent tissue in order to gain a better understanding of colonisation patterns in CRC patients. By quantifying multiple CRC-associated bacteria in one cohort, we have been able to uncover inter- and intra-individual patterns of colonisation not previously recognised. We further identified significant associations with clinicopathological features including MSI-H (*Fusobacterium*), stage of disease (ETBF and *Fusobacterium*), tumour location (ETBF and *afaC*-positive *E*. *coli*), age (*Fusobacterium*), as well as a positive association between *Fusobacterium* and *pks*-positive strains.

Notably, our finding that late stage (III) tumour samples were significantly associated with high-level colonisation by *Fusobacterium* is consistent with previous studies demonstrating a positive association between high-level colonisation by *Fusobacterium* and regional lymph node metastases [5,45]. Bonnet *et al*. found a similar trend between cyclomodulin-positive *E*. *coli*, and stage III/IV colon cancers, which we however did not observe here [52]. Tumour tissue provides a nutrient-rich surface that is not protected by an intact mucosal layer, and the tumour-homing activity of certain bacteria is well documented [53], but this does not necessarily imply oncogenic potential. However, in addition to the enrichment of *Fusobacterium* in tumour vs. normal tissues and in late stage CRCs, *Fusobacterium* spp. are also enriched in irritable bowel disease (IBD) patients (who have a 2–3 fold increased risk of developing CRC) [54] compared to healthy controls. Interestingly, *Fusobacterium* spp. isolated from inflamed tissue in IBD patients were significantly more invasive in a subsequent *in vitro* assay compared to non-inflamed tissue from IBD patients or healthy controls [55], possibly suggesting an active role for *Fusobacterium* in gastrointestinal diseases.

Our finding that *Fusobacterium* levels positively correlate with MSI-H, younger age and black ethnicity may be particularly relevant in the South African setting where a disproportionately high number of *young* black CRC patients––41–57% of black CRC patients [56–58] compared to only 10% of white CRC patients under the age of 50 [56]–has been reported. CRCs in young black patients do not appear to originate from colonic polyps [57] and have not been linked to IBD or diverticulosis [57]; the majority of these cancers are located in the proximal colon [56–58] (often in the caecum [57]), and often display mucinous histology [58,59] and a higher rate of microsatellite instability (MSI) compared to older patients [56,59]. Our findings may therefore suggest a genetic aspect to susceptibility to high-level colonisation by *Fusobacterium*, which warrants further investigation in a larger cohort.

Tahara *et al*. have previously observed an association between high-level colonisation by *Fusobacterium* and MSI-H, *MLH1* methylation as well as the CpG island methylator phenotype (CIMP) [45] suggesting that *Fusobacterium* might promote MSI by inducing *MLH1* hypermethylation. Importantly, the association between MSI and *Fusobacterium* observed here was independent of the origin of MSI in our cohort, with 4/8 HNPCC adenocarcinoma samples falling into the *Fusobacterium*-high group of colonisation. HNPCC requires inactivation of both alleles of the affected mismatch-repair gene and it is possible that *Fusobacterium* precipitates loss of the wild-type allele through methylation. However, the role of aberrant methylation in the aetiopathogenesis of HNPCC remains questionable: Kaz *et al*. found promoter methylation of *MLH1* in 53% of HNPCC adenomas [60], compared to only 4% of sporadic adenomas, whilst Speake *et al*. found 40% and 25% of hyperplastic polyps of sporadic or HNPCC origin to be CIMP-H [61]. However, LOH or gene conversion are the most frequent mechanism of inactivation of the wild type *MLH1* allele in HNPCC tumours [62–66]. Further, HNPCC and sporadic MSI-H cancers have distinct histological and molecular features: While, both cancer types display lymphocytic infiltration, mucin secretion and poor differentiation [67], HNPCCs tends to originate from classical adenomas compared to sessile-serrated adenomas in the case of MSI-H CRCs [68]; on a molecular level, HNPCCs are strongly associated with mutations in *APC* or ß-catenin and/or *KRAS* [67,68], while MSI-H sporadic CRCs instead exhibit *BRAF* mutations, which are present in CRC precursor lesions [68]. Therefore, while it is possible that *Fusobacterium* might cause MSI (and thereby CRC), it seems more likely that *Fusobacterium* preferentially flourishes in MSI-H compared to MSS cancers, perhaps due to the altered glycosylation profile in MSI-H cancers [69], that could facilitate adherence of certain bacteria [70]. Additionally, *F*. *nucleatum* infection has been shown to stimulate proliferation in CRC- but not in non-neoplastic-cell lines [16] and, *Fusobacterium* spp. have been demonstrated to stimulate cellular proliferation following an initial oncogenic hit (affecting a component of the WNT signaling pathway) in mice [71] and in CRC cell lines [16]. Taken together, it therefore seems most likely that *Fusobacterium* is not oncogenic itself, but may contribute to tumourigenesis by promoting inflammation and cancer cell proliferation.

It has long been appreciated that certain individuals are more susceptible to aberrant pathogenic colonization of the gut epithelium, which may be accompanied by chronic inflammation, for example in patients with IBD. However, our finding that colonisation by certain bacteria are significantly associated with clinicopathological features in CRC—including the stage and site of disease—is new and might be linked to differential susceptibilities in relation to clinical features, such as age and ethnicity; these association do not necessarily imply oncogenicity since many of the CRC-associated bacteria investigated in this study are asymptomatically present in a significant percentage of the population [12,46]. One might therefore expect a pathogenic trend similar to that of *H*. *pylori* where genetic, environmental and strain-specific risk modifiers govern susceptibility to bacterially-mediated oncogenesis in the colon and where only a small fraction of individuals infected with the bacterium will eventually develop cancer. Evaluating the distribution of bacteria in relation to ethnicity, lifestyle- and clinicopathological factors is the first step in evaluating host-susceptibility to infection and putative bacterially-mediated oncogenic mechanisms. Furthermore, bacterial abundance is not the only factor that may be correlated with clinicopathological features since low-abundant bacteria may exert a significant effect on the host through the secretion of toxins at high levels. For example, Dutilh *et al*. showed that Enterobacterial toxins were among the most highly expressed in metatranscriptomic sequencing data from CRC paired tumour and normal tissues [72], including toxins from *E*. *coli*, *Salmonella enterica* and *Shigella flexneri* [72]. Evaluating the presence of bacterial toxins with oncogenic potential at the transcriptional or proteomic level will thus provide an additional layer of information to unravel complex host-pathogen interactions with relevance to CRC in the future. Future studies should also be aimed at validating our findings in a larger cohort (particularly in MSI-H CRCs in the case of EPEC); and at profiling *Fusobacterium* at the species level, as well as other AIEC toxins implicated in CRC not examined here, such as *lpfA*.

Establishing causality for any of the bacteria examined here remains a challenge and would require rigorous investigation in animal models as well as large scale epidemiological data, as was used in establishing causality in the case of *H*. *pylori* and gastric cancer. However, by evaluating the distribution of bacteria in relation to ethnicity, lifestyle- and clinicopathological factors in a South African cohort, we have taken a first step towards this goal and we expect that our data will facilitates the development of targeted research questions for future studies.

## Supporting Information

S1 Table Participant-level characteristics. (a). FF = fresh-frozen; FFPE = formalin-fixed paraffin embedded; Tissue type: N = matched normal mucosa, T = tumour tissue; Ethnicity: MA = mixed ancestry, C = caucasian, B = black, I = indian; Gender: M = male, F = female; Stage: Dukes stage of tumour tissue. (b). Bacterial quantitation data expressed as bacteria/50ng human DNA; EPEC limit of detection (LOD) & *Fusobacterium* LOD: for FFPE tissue these are the estimated LOD′s based on normalisation against COX1; MSI method: PCR = Bethesda panel of markers; MLH1 meth. = MLH1 methylation testing by methylation-specific PCR; MMR prot. = MMR protein(s) with known methylation or absence of staining by immunohistochemistry (IHC) of MLH1, MSH2 and MSH6; FB = *Fusobacterium*; EF = *E*. *faecalis*.
(DOCX)

S2 Table Primers and their limits of detection (LODs) and qPCR efficiencies. (DOCX)

S3 Table qPCR conditions used. (DOCX)
