# Supplementary material for: Quantitative Profiling of Colorectal Cancer-Associated Bacteria Reveals Associations between Fusobacterium spp., Enterotoxigenic Bacteroides fragilis (ETBF) and Clinicopathological Features of Colorectal Cancer
Source: PLoS One. 2015 Mar 9;10(3):e0119462. doi: 10.1371/journal.pone.0119462 (PMC4353626; doi:10.1371/journal.pone.0119462)
Supplement: S1 Table — (a). FF = fresh-frozen; FFPE = formalin-fixed paraffin embedded; Tissue type: N = matched normal mucosa, T = tumour tissue; Ethnicity: MA = mixed ancestry, C = caucasian, B = black, I = indian; Gender: M = male, F = female; Stage: Dukes stage of tumour tissue. (b). Bacterial quantitation data expressed as bacteria/50ng human DNA; EPEC limit of detection (LOD) & Fusobacterium LOD: for FFPE tissue these are the estimated LOD′s based on normalisation against COX1; MSI method: PCR = Bethesda panel of markers; MLH1 meth. = MLH1 methylation testing by methylation-specific PCR; MMR prot. = MMR protein(s) with known methylation or absence of staining by immunohistochemistry (IHC) of MLH1, MSH2 and MSH6; FB = Fusobacterium; EF = E. faecalis. (DOCX) [file pone.0119462.s001.docx]

Table S1: Participant-level characteristics table

(a) FF = fresh-frozen; FFPE = formalin-fixed paraffin embedded; Tissue type: N=matched normal mucosa, T = tumour tissue; Ethnicity: MA = mixed ancestry, C = caucasian, B = black, I = indian; Gender: M = male, F = female; Stage: Dukes stage of tumour tissue.

| **Sample** | **Specimen Type** | **Tumour Type** | **Tissue type** | **Patient** | **Age** | **Ethnicity** | **RT** | **Gender** | **BMI** | **Stage** | **Location** | **Site** |
| --- | --- | --- | --- | --- | --- | --- | --- | --- | --- | --- | --- | --- |
| 10N | FF | Sporadic | N | 10 | 63 | MA | N | M | 24.7 | NA | Distal | Descending colon |
| 10T | FF | Sporadic | T | 10 | 63 | MA | N | M | 24.7 | III | Distal | Descending colon |
| 11N | FF | Sporadic | N | 11 | 84 | C | N | M | 28.7 | NA | Proximal | Transverse colon |
| 11T | FF | Sporadic | T | 11 | 84 | C | N | M | 28.7 | I | Proximal | Transverse colon |
| 13N | FF | HNPCC | N | 13 | 46 | MA | N | F | 22.3 | NA | Proximal | Ceacum |
| 13T | FF | HNPCC | T | 13 | 46 | MA | N | F | 22.3 | II | Proximal | Ceacum |
| 14N | FF | Sporadic | N | 14 | 80 | MA | N | M | 23.5 | NA | Distal | Sigmoid colon |
| 14T | FF | Sporadic | T | 14 | 80 | MA | N | M | 23.5 | IV | Distal | Sigmoid colon |
| 15N | FF | Sporadic | N | 15 | 74 | I | N | M | 24.2 | NA | Distal | Rectum |
| 15T | FF | Sporadic | T | 15 | 74 | I | N | M | 24.2 | II | Distal | Rectum |
| 16N | FF | Sporadic | N | 16 | 76 | C | N | M | NA | NA | Proximal | Ascending colon |
| 16T | FF | Sporadic | T | 16 | 76 | C | N | M | NA | I | Proximal | Ascending colon |
| 17N | FF | Sporadic | N | 17 | 79 | C | N | M | 22.7 | NA | Distal | Sigmoid colon |
| 17T | FF | Sporadic | T | 17 | 79 | C | N | M | 22.7 | III | Distal | Sigmoid colon |
| 18N | FF | HNPCC | N | 18 | 44 | MA | N | F | NA | NA | Proximal | Transverse colon |
| 18T | FF | HNPCC | T | 18 | 44 | MA | N | F | NA | I | Proximal | Transverse colon |
| 1N | FF | HNPCC | N | 1 | 58 | MA | N | F | 29.6 | NA | Proximal | Transverse colon |
| 1T | FF | HNPCC | T | 1 | 58 | MA | N | F | 29.6 | IV | Proximal | Transverse colon |
| 20N | FF | HNPCC | N | 20 | NA | MA | N | F | NA | NA | Proximal | Ceacum |
| 20T | FF | HNPCC | T | 20 | NA | MA | N | F | NA | NA | Proximal | Ceacum |
| 23N | FF | Sporadic | N | 23 | 70 | MA | N | F | NA | NA | Distal | Splenic flexure |
| 23T | FF | Sporadic | T | 23 | 70 | MA | N | F | NA | III | Distal | Splenic flexure |
| 33N | FF | Sporadic | N | 33 | 69 | MA | N | F | 28.7 | NA | Distal | Rectum |
| 33T | FF | Sporadic | T | 33 | 69 | MA | N | F | 28.7 | III | Distal | Rectum |
| 34N | FF | Sporadic | N | 34 | 70 | MA | N | F | 25.4 | NA | Distal | Rectum |
| 34T | FF | Sporadic | T | 34 | 70 | MA | N | F | 25.4 | III | Distal | Rectum |
| 37N | FF | Sporadic | N | 37 | 49 | B | N | M | 20 | NA | Distal | Proximal descending colon |
| 37T | FF | Sporadic | T | 37 | 49 | B | N | M | 20 | III | Distal | Proximal descending colon |
| 3N | FF | Sporadic | N | 3 | 70 | MA | N | M | 29.5 | NA | Distal | RSJ |
| 3T | FF | Sporadic | T | 3 | 70 | MA | N | M | 29.5 | III | Distal | RSJ |
| 41N | FF | Sporadic | N | 41 | 71 | MA | N | F | 31.6 | NA | Distal | Rectum |
| 41T | FF | Sporadic | T | 41 | 71 | MA | N | F | 31.6 | II | Distal | Rectum |
| 44N | FF | Sporadic | N | 44 | 36 | B | N | M | 18.2 | NA | Proximal | Ceacum |
| 44T | FF | Sporadic | T | 44 | 36 | B | N | M | 18.2 | III | Proximal | Ceacum |
| 48N | FF | Sporadic | N | 48 | 37 | MA | N | M | 30.7 | NA | Distal | NA |
| 48T | FF | Sporadic | T | 48 | 37 | MA | N | M | 30.7 | IV | Distal | NA |
| 4N | FF | HNPCC | N | 4 | 44 | MA | N | F | 26.7 | NA | Proximal | Ceacum |
| 4T | FF | HNPCC | T | 4 | 44 | MA | N | F | 26.7 | III | Proximal | Ceacum |
| 55N | FF | Sporadic | N | 55 | 64 | MA | N | M | 25.8 | NA | Distal | NA |
| 55T | FF | Sporadic | T | 55 | 64 | MA | N | M | 25.8 | III | Distal | NA |
| 56N | FF | Sporadic | N | 56 | 54 | MA | N | F | 26.9 | NA | Distal | RSJ |
| 56T | FF | Sporadic | T | 56 | 54 | MA | N | F | 26.9 | III | Distal | RSJ |
| 60N | FF | Sporadic | N | 60 | 65 | MA | N | M | NA | NA | Distal | RSJ |
| 60T | FF | Sporadic | T | 60 | 65 | MA | N | M | NA | II | Distal | RSJ |
| 63N | FF | Sporadic | N | 63 | 78 | C | N | F | 26.8 | NA | Proximal | Hepatic flexure |
| 63T | FF | Sporadic | T | 63 | 78 | C | N | F | 26.8 | II | Proximal | Hepatic flexure |
| 8N | FF | Sporadic | N | 8 | 61 | C | N | M | 25 | NA | Distal | Rectum |
| 8T | FF | Sporadic | T | 8 | 61 | C | N | M | 25 | III | Distal | Rectum |
| 19N | FF | Sporadic | N | 19 | 62 | MA | Y | F | NA | NA | Distal | Rectum |
| 19T | FF | Sporadic | T | 19 | 62 | MA | Y | F | NA | I | Distal | Rectum |
| 21N | FF | Sporadic | N | 21 | 61 | MA | N | F | 40.5 | NA | Distal | Rectum |
| 21T | FF | Sporadic | T | 21 | 61 | MA | N | F | 40.5 | NA | Distal | Rectum |
| 24N | FF | Sporadic | N | 24 | 42 | MA | Y | M | NA | NA | Distal | Rectum |
| 24T | FF | Sporadic | T | 24 | 42 | MA | Y | M | NA | III | Distal | Rectum |
| 25N | FF | Sporadic | N | 25 | 67 | MA | Y | F | NA | NA | Distal | Rectum |
| 25T | FF | Sporadic | T | 25 | 67 | MA | Y | F | NA | I | Distal | Rectum |
| 26N | FF | Sporadic | N | 26 | 73 | C | N | F | NA | NA | Distal | Rectum |
| 26T | FF | Sporadic | T | 26 | 73 | C | N | F | NA | I | Distal | Rectum |
| 28N | FF | Sporadic | N | 28 | 79 | I | Y | M | NA | NA | Distal | Rectum |
| 28T | FF | Sporadic | T | 28 | 79 | I | Y | M | NA | II | Distal | Rectum |
| 29N | FF | Sporadic | N | 29 | 73 | B | Y | M | 25.8 | NA | Distal | Rectum |
| 29T | FF | Sporadic | T | 29 | 73 | B | Y | M | 25.8 | III | Distal | Rectum |
| 2N | FF | Sporadic | N | 2 | 67 | MA | Y | M | NA | NA | Distal | Rectum |
| 2T | FF | Sporadic | T | 2 | 67 | MA | Y | M | NA | II | Distal | Rectum |
| 30N | FF | Sporadic | N | 30 | 25 | B | Y | M | NA | NA | Distal | Rectum |
| 30T | FF | Sporadic | T | 30 | 25 | B | Y | M | NA | II | Distal | Rectum |
| 35N | FF | Sporadic | N | 35 | 68 | MA | Y | M | NA | NA | Distal | Rectum |
| 35T | FF | Sporadic | T | 35 | 68 | MA | Y | M | NA | II | Distal | Rectum |
| 39N | FF | Sporadic | N | 39 | 51 | MA | Y | F | 36.3 | NA | Distal | Rectum |
| 39T | FF | Sporadic | T | 39 | 51 | MA | Y | F | 36.3 | II | Distal | Rectum |
| 45N | FF | Sporadic | N | 45 | 36 | B | Y | F | NA | NA | Distal | Rectum |
| 45T | FF | Sporadic | T | 45 | 36 | B | Y | F | NA | II | Distal | Rectum |
| 47N | FF | HNPCC | N | 47 | 25 | MA | Y | M | NA | NA | Distal | Descending colon |
| 47T | FF | HNPCC | T | 47 | 25 | MA | Y | M | NA | III | Distal | Descending colon |
| 54N | FF | Sporadic | N | 54 | 60 | MA | N | M | NA | NA | NA | NA |
| 54T | FF | Sporadic | T | 54 | 60 | MA | N | M | NA | NA | NA | NA |
| 58N | FF | Sporadic | N | 58 | 71 | MA | Y | NA | NA | NA | Distal | Rectum |
| 58T | FF | Sporadic | T | 58 | 71 | MA | Y | NA | NA | NA | Distal | Rectum |
| 61N | FF | Sporadic | N | 61 | 61 | MA | N | F | NA | NA | Distal | Sigmoid colon |
| 61T | FF | Sporadic | T | 61 | 61 | MA | N | F | NA | NA | Distal | Sigmoid colon |
| 62N | FF | Sporadic | N | 62 | 40 | MA | Y | F | NA | NA | Distal | Rectum |
| 62T | FF | Sporadic | T | 62 | 40 | MA | Y | F | NA | II | Distal | Rectum |
| 65N | FF | Sporadic | N | 65 | 73 | C | Y | M | NA | NA | Distal | Rectum |
| 65T | FF | Sporadic | T | 65 | 73 | C | Y | M | NA | III | Distal | Rectum |
| 66N | FF | Sporadic | N | 66 | 59 | MA | Y | F | NA | NA | Distal | Rectum |
| 66T | FF | Sporadic | T | 66 | 59 | MA | Y | F | NA | III | Distal | Rectum |
| 67N | FF | Sporadic | N | 67 | 64 | MA | N | M | 23.8 | NA | Distal | Rectum |
| 67T | FF | Sporadic | T | 67 | 64 | MA | N | M | 23.8 | III | Distal | Rectum |
| 69N | FF | Sporadic | N | 69 | NA | NA | NA | F | NA | NA | NA | NA |
| 69T | FF | Sporadic | T | 69 | NA | NA | NA | F | NA | NA | NA | NA |
| 6N | FF | Sporadic | N | 6 | 23 | B | Y | F | NA | NA | Distal | Rectum |
| 6T | FF | Sporadic | T | 6 | 23 | B | Y | F | NA | III | Distal | Rectum |
| 7N | FF | Sporadic | N | 7 | 52 | MA | N | M | 27.9 | NA | Distal | Rectum |
| 7T | FF | Sporadic | T | 7 | 52 | MA | N | M | 27.9 | III | Distal | Rectum |
| 22N | FF | Sporadic | N | 22 | 49 | MA | Y | M | NA | NA | Distal | Rectum |
| 22T | FF | Sporadic | T | 22 | 49 | MA | Y | M | NA | III | Distal | Rectum |
| 40N | FF | Sporadic | N | 40 | 47 | MA | Y | F | NA | NA | Distal | Rectum |
| 40T | FF | Sporadic | T | 40 | 47 | MA | Y | F | NA | II | Distal | Rectum |
| 43N | FF | Sporadic | N | 43 | 67 | MA | Y | F | NA | NA | Distal | Rectum |
| 43T | FF | Sporadic | T | 43 | 67 | MA | Y | F | NA | II | Distal | Rectum |
| 51N | FF | Sporadic | N | 51 | 47 | MA | Y | M | NA | NA | Distal | Rectum |
| 51T | FF | Sporadic | T | 51 | 47 | MA | Y | M | NA | II | Distal | Rectum |
| 57N | FF | Sporadic | N | 57 | 45 | MA | N | M | 25.7 | NA | Distal | RSJ |
| 57T | FF | Sporadic | T | 57 | 45 | MA | N | M | 25.7 | III | Distal | RSJ |
| 59N | FF | Sporadic | N | 59 | 79 | C | Y | F | NA | NA | NA | NA |
| 59T | FF | Sporadic | T | 59 | 79 | C | Y | F | NA | II | NA | NA |
| 64N | FF | Sporadic | N | 64 | 69 | MA | Y | F | NA | NA | Distal | Rectum |
| 64T | FF | Sporadic | T | 64 | 69 | MA | Y | F | NA | II | Distal | Rectum |
| 31N | FF | Sporadic | N | 31 | 54 | MA | Y | F | NA | NA | Distal | Rectum |
| 31T | FF | Sporadic | T | 31 | 54 | MA | Y | F | NA | II | Distal | Rectum |
| 71T | FFPE | Sporadic | T | 71 | 41 | NA | N | F | NA | II | Proximal | Hepatic flexure |
| 71N | FFPE | Sporadic | N | 71 | 41 | NA | N | F | NA | NA | Proximal | Hepatic flexure |
| 72T | FFPE | Sporadic | T | 72 | 43 | B | N | M | NA | III | Proximal | Ceacum |
| 72N | FFPE | Sporadic | N | 72 | 43 | B | N | M | NA | NA | Proximal | Ceacum |
| 73T | FFPE | Sporadic | T | 73 | 30 | NA | N | F | NA | III | Proximal | Ceacum |
| 73N | FFPE | Sporadic | N | 73 | 30 | NA | N | F | NA | NA | Proximal | Ceacum |
| 74T | FFPE | Sporadic | T | 74 | 41 | NA | Y | F | NA | IV | Distal | Rectum |
| 74N | FFPE | Sporadic | N | 74 | 41 | NA | Y | F | NA | NA | Distal | Rectum |
| 75T | FFPE | Sporadic | T | 75 | NA | NA | NA | NA | NA | NA | NA | NA |
| 76T | FFPE | Sporadic | T | 76 | 41 | B | N | M | NA | II | Proximal | Ceacum |
| 76N | FFPE | Sporadic | N | 76 | 41 | B | N | M | NA | NA | Proximal | Ceacum |
| 77T | FFPE | Sporadic | T | 77 | NA | NA | NA | NA | NA | NA | NA | NA |
| 78T | FFPE | Sporadic | T | 78 | NA | NA | NA | NA | NA | NA | NA | NA |
| 79T | FFPE | HNPCC | T | 79 | NA | NA | NA | NA | NA | NA | NA | NA |
| 80T | FFPE | Sporadic | T | 80 | NA | NA | NA | NA | NA | NA | NA | NA |
| 81T | FFPE | Sporadic | T | 81 | NA | NA | NA | NA | NA | III | NA | NA |
| 82T | FFPE | Sporadic | T | 82 | NA | NA | NA | NA | NA | NA | NA | NA |
| 83T | FFPE | Sporadic | T | 83 | NA | NA | NA | NA | NA | NA | NA | NA |
| 84T | FFPE | Sporadic | T | 84 | NA | NA | NA | NA | NA | NA | NA | NA |
| 85T | FFPE | Sporadic | T | 85 | 35 | NA | N | M | NA | III | Distal | Sigmoid colon |
| 85N | FFPE | Sporadic | N | 85 | 35 | NA | N | M | NA | NA | Distal | Sigmoid colon |
| 86T | FFPE | Sporadic | T | 86 | NA | NA | NA | NA | NA | NA | NA | NA |
| 87T | FFPE | Sporadic | T | 87 | NA | NA | NA | NA | NA | NA | NA | NA |
| 88T | FFPE | HNPCC | T | 88 | NA | NA | NA | NA | NA | NA | NA | NA |

(b) Participant characteristics, table 2/2. Bacterial quantitation data expressed as bacteria/50ng human DNA; EPEC limit of detection (LOD) & *Fusobacterium* LOD: for FFPE tissue these are the estimated LOD's based on normalisation against COX1; MSI method: PCR = Bethesda panel of markers; MLH1 meth. = MLH1 methylation testing by methylation-specific PCR; MMR prot. = MMR protein(s) with known methylation or absence of staining by immunohistochemistry (IHC) of MLH1, MSH2 and MSH6; FB = *Fusobacterium*; EF = *E. faecalis*.

| **Sample** | **MSI** | **MSI method** | **MLH1 meth.** | **MMR prot.** | **Inflammation noted (pathology report)** | **ETBF** | **EPEC** | **EPEC LOD** | **EF** | **FB** | **FB LOD** | **afaC+ *E. coli*** | **ClB+ *E. coli*** |
| --- | --- | --- | --- | --- | --- | --- | --- | --- | --- | --- | --- | --- | --- |
| 10N | MSS | PCR | NA | NA | N | 12 | 0 | 10 | NA | 8 | 2 | 2177 | 0 |
| 10T | MSS | PCR | NA | NA | N | 139 | 0 | 10 | 0 | 1 | 2 | 170 | 0 |
| 11N | MSS | PCR | N | NA | N | 0 | 0 | 10 | 0 | 0 | 2 | 0 | 0 |
| 11T | MSS | PCR | N | NA | N | 0 | 0 | 10 | 0 | 0 | 2 | 0 | 0 |
| 13N | MSI-H | PCR | N | NA | Y | 374 | 0 | 10 | 0 | 65 | 2 | 3787 | 0 |
| 13T | MSI-H | PCR | N | MLH1 | Y | 3186 | 0 | 10 | 1 | 3273 | 2 | 19200 | 0 |
| 14N | MSS | PCR | N | NA | N | 3423 | 0 | 10 | 331 | 67 | 2 | 261 | 0 |
| 14T | MSS | PCR | N | NA | N | 987 | 0 | 10 | 151 | 271 | 2 | 729 | 0 |
| 15N | MSS | PCR | N | NA | N | 2 | 0 | 10 | 0 | 9 | 2 | 4787 | 1172 |
| 15T | MSS | PCR | N | NA | N | 0 | 0 | 10 | 0 | 17 | 2 | 9887 | 1707 |
| 16N | MSS | PCR | N | NA | Y | 0 | 0 | 10 | 0 | 1953 | 2 | 319000 | 1780 |
| 16T | MSS | PCR | N | NA | Y | 0 | 0 | 10 | 0 | 21 | 2 | 2043 | 0 |
| 17N | MSS | PCR | N | NA | N | 130 | 0 | 10 | 0 | 48 | 2 | 15 | 0 |
| 17T | MSS | PCR | N | NA | N | 486 | 0 | 10 | 0 | 833 | 2 | 81 | 0 |
| 18N | MSI-H | PCR | N | NA | NA | 0 | 0 | 10 | 5 | 377 | 2 | 1937 | 0 |
| 18T | MSI-H | PCR | N | NA | NA | 0 | 0 | 10 | 3 | 2610 | 2 | 7593 | 994 |
| 1N | MSS | PCR | N | NA | N | 4 | 0 | 10 | 0 | 12 | 2 | 12 | 0 |
| 1T | MSS | PCR | N | NA | N | 2 | 0 | 10 | 0 | 276 | 2 | 47 | 0 |
| 20N | MSI-H | PCR | N | NA | NA | 3 | 0 | 10 | NA | 0 | 2 | 0 | 0 |
| 20T | MSI-H | PCR | N | NA | NA | 13 | 0 | 10 | NA | 2 | 2 | 0 | 0 |
| 23N | MSS | PCR | N | NA | Y | 5 | 0 | 10 | 1 | 26 | 2 | 0 | 0 |
| 23T | MSS | PCR | N | NA | Y | 3 | 0 | 10 | 250 | 4820 | 2 | 0 | 0 |
| 33N | MSS | PCR | N | NA | N | 0 | 0 | 10 | 0 | 3 | 2 | 0 | 677 |
| 33T | MSS | PCR | N | NA | N | 0 | 0 | 10 | 0 | 14 | 2 | 0 | 622 |
| 34N | MSS | PCR | N | NA | N | 0 | 52 | 10 | 0 | 25 | 2 | 30900 | 1263 |
| 34T | MSS | PCR | N | NA | N | 3 | 63 | 10 | 1 | 289 | 2 | 33433 | 1400 |
| 37N | MSS | PCR | N | NA | Y | 1620 | 0 | 10 | NA | 1897 | 2 | 0 | 3693 |
| 37T | MSS | PCR | N | NA | Y | 35300 | 0 | 10 | 0 | 4773 | 2 | 0 | 1800 |
| 3N | MSS | PCR | N | NA | N | 0 | 0 | 10 | 0 | 1 | 2 | 0 | 0 |
| 3T | MSS | PCR | N | NA | N | 0 | 0 | 10 | 0 | 3 | 2 | 0 | 0 |
| 41N | MSS | PCR | N | NA | N | 0 | 0 | 10 | NA | 69 | 2 | 0 | 0 |
| 41T | MSS | PCR | N | NA | N | 0 | 0 | 10 | NA | 213 | 2 | 2 | 0 |
| 44N | MSI-H | PCR | N | NA | Y | 0 | 3037 | 10 | 0 | 1110 | 2 | 1197 | 36 |
| 44T | MSI-H | PCR | Y | NA | Y | 0 | 1111 | 10 | 0 | 68700 | 2 | 156 | 0 |
| 48N | MSS | PCR | N | NA | N | 2328 | 0 | 10 | 0 | 1 | 2 | 0 | 0 |
| 48T | MSS | PCR | N | NA | N | 1106 | 0 | 10 | 0 | 44 | 2 | 0 | 0 |
| 4N | MSI-H | PCR | N | NA | N | 0 | 0 | 10 | 0 | 20 | 2 | 195 | 0 |
| 4T | MSI-H | PCR | N | NA | N | 3 | 0 | 10 | 0 | 60767 | 2 | 6537 | 0 |
| 55N | MSI-L | PCR | N | NA | N | 4 | 0 | 10 | 0 | 99 | 2 | 0 | 5777 |
| 55T | MSI-L | PCR | N | NA | N | 3 | 0 | 10 | 0 | 9 | 2 | 0 | 536 |
| 56N | MSS | PCR | N | NA | N | 698 | 0 | 10 | 0 | 46 | 2 | 0 | 0 |
| 56T | MSS | PCR | N | NA | N | 648 | 0 | 10 | 0 | 378 | 2 | 0 | 0 |
| 60N | MSI-H | PCR | N | NA | N | 20 | 0 | 10 | 0 | 3 | 2 | 2750 | 78 |
| 60T | MSI-H | PCR | N | NA | N | 0 | 0 | 10 | 0 | 22 | 2 | 5237 | 69 |
| 63N | MSI-H | PCR | N | NA | N | 0 | 0 | 10 | NA | 73 | 2 | 0 | 0 |
| 63T | MSI-H | PCR | Y | NA | N | 0 | 13 | 10 | 4 | 2730 | 2 | 0 | 0 |
| 8N | MSS | PCR | N | NA | N | 12 | 0 | 10 | 4 | 0 | 2 | 592 | 0 |
| 8T | MSS | PCR | N | NA | N | 14 | 0 | 10 | 2 | 37 | 2 | 619 | 0 |
| 19N | NA | NA | NA | NA | NA | 0 | 0 | 10 | 0 | 0 | 2 | 0 | 0 |
| 19T | NA | NA | NA | NA | NA | 0 | 0 | 10 | 0 | 0 | 2 | 0 | 0 |
| 21N | MSS | PCR | NA | NA | NA | 0 | 0 | 10 | NA | 0 | 2 | 0 | 0 |
| 21T | MSS | PCR | N | NA | NA | 0 | 0 | 10 | NA | 0 | 2 | 1 | 0 |
| 24N | NA | NA | NA | NA | NA | 0 | 0 | 10 | 0 | 21 | 2 | 0 | 0 |
| 24T | NA | NA | NA | NA | NA | 0 | 0 | 10 | NA | 57 | 2 | 0 | 0 |
| 25N | NA | NA | NA | NA | NA | 0 | 0 | 10 | 0 | 8 | 2 | 0 | 0 |
| 25T | NA | NA | NA | NA | NA | 0 | 0 | 10 | 0 | 0 | 2 | 0 | 0 |
| 26N | MSI-L | PCR | N | NA | N | 0 | 0 | 10 | 4 | 333 | 2 | 0 | 34 |
| 26T | MSI-L | PCR | N | NA | N | 0 | 0 | 10 | NA | 40 | 2 | 0 | 0 |
| 28N | NA | NA | NA | NA | NA | 0 | 0 | 10 | 0 | 1 | 2 | 0 | 0 |
| 28T | NA | NA | NA | NA | NA | 0 | 0 | 10 | 0 | 0 | 2 | 0 | 0 |
| 29N | MSS | PCR | NA | NA | N | 0 | 14 | 10 | 0 | 69 | 2 | 0 | 0 |
| 29T | MSS | PCR | NA | NA | N | 0 | 31 | 10 | 0 | 6678 | 2 | 19 | 0 |
| 2N | NA | NA | NA | NA | NA | 0 | 0 | 10 | 0 | 71 | 2 | 0 | 0 |
| 2T | NA | NA | NA | NA | NA | 0 | 0 | 10 | 0 | 1026 | 2 | 0 | 0 |
| 30N | NA | NA | NA | NA | NA | 0 | 0 | 10 | 4 | 1543 | 2 | 0 | 22800 |
| 30T | NA | NA | NA | NA | NA | 0 | 0 | 10 | 0 | 106 | 2 | 0 | 0 |
| 35N | NA | NA | NA | NA | NA | 0 | 0 | 10 | 0 | 422 | 2 | 0 | 0 |
| 35T | NA | NA | NA | NA | NA | 0 | 0 | 10 | 1 | 1353 | 2 | 0 | 0 |
| 39N | NA | NA | NA | NA | N | 0 | 0 | 10 | NA | 11 | 2 | 0 | 0 |
| 39T | NA | NA | NA | NA | N | 0 | 0 | 10 | NA | 49 | 2 | 0 | 0 |
| 45N | NA | NA | NA | NA | NA | 0 | 0 | 10 | 0 | 0 | 2 | 0 | 0 |
| 45T | NA | NA | NA | NA | NA | 0 | 51 | 10 | 0 | 5 | 2 | 0 | 0 |
| 47N | NA | NA | NA | NA | Y | 5 | 0 | 10 | 0 | 187 | 2 | 0 | 0 |
| 47T | NA | NA | NA | NA | Y | 0 | 0 | 10 | 0 | 125 | 2 | 0 | 0 |
| 54N | MSS | PCR | N | NA | NA | 0 | 0 | 10 | 0 | 0 | 2 | 0 | 516 |
| 54T | MSS | PCR | N | NA | NA | 0 | 0 | 10 | 0 | 8 | 2 | 0 | 316 |
| 58N | NA | NA | NA | NA | NA | 0 | 0 | 10 | 0 | 1 | 2 | 2 | 0 |
| 58T | NA | NA | NA | NA | NA | 0 | 0 | 10 | 0 | 0 | 2 | 2 | 0 |
| 61N | MSI-L | PCR | N | NA | Y | 0 | 0 | 10 | 0 | 36 | 2 | 25 | 0 |
| 61T | MSI-L | PCR | N | NA | Y | 0 | 0 | 10 | 0 | 14 | 2 | 20 | 60 |
| 62N | NA | NA | NA | NA | NA | 0 | 0 | 10 | NA | 5 | 2 | 0 | 0 |
| 62T | NA | NA | NA | NA | NA | 0 | 0 | 10 | NA | 78 | 2 | 0 | 0 |
| 65N | NA | NA | NA | NA | NA | 0 | 0 | 10 | 8 | 340 | 2 | 10 | 3617 |
| 65T | NA | NA | NA | NA | NA | 0 | 0 | 10 | 131 | 1353 | 2 | 0 | 3497 |
| 66N | NA | NA | N | NA | NA | 0 | 0 | 10 | 0 | 38 | 2 | 0 | 0 |
| 66T | NA | NA | N | NA | NA | 0 | 0 | 10 | 1 | 8990 | 2 | 0 | 0 |
| 67N | MSS | PCR | N | NA | N | 0 | 0 | 10 | NA | 217 | 2 | 0 | 0 |
| 67T | MSS | PCR | N | NA | N | 0 | 0 | 10 | 12 | 1483 | 2 | 0 | 0 |
| 69N | NA | NA | NA | NA | NA | 0 | 0 | 10 | 0 | 0 | 2 | 29 | 0 |
| 69T | NA | NA | NA | NA | NA | 0 | 0 | 10 | 0 | 240 | 2 | 23 | 0 |
| 6N | MSS | PCR | NA | NA | NA | 0 | 0 | 10 | 0 | 273 | 2 | 0 | 0 |
| 6T | MSS | PCR | NA | NA | NA | 0 | 0 | 10 | 0 | 2417 | 2 | 0 | 0 |
| 7N | MSS | PCR | N | NA | N | 0 | 0 | 10 | 0 | 1397 | 2 | 0 | 44 |
| 7T | MSS | PCR | N | NA | N | 0 | 0 | 10 | 0 | 8497 | 2 | 0 | 62 |
| 22N | NA | NA | NA | NA | NA | 0 | 0 | 10 | NA | 4 | 2 | 0 | 0 |
| 22T | NA | NA | NA | NA | NA | 0 | 41 | 10 | NA | 5740 | 2 | 0 | 7373 |
| 40N | NA | NA | NA | NA | NA | 0 | 0 | 10 | NA | 5 | 2 | 0 | 0 |
| 40T | NA | NA | NA | NA | NA | 0 | 0 | 10 | NA | 3 | 2 | 0 | 0 |
| 43N | NA | NA | NA | NA | NA | NA | 0 | 10 | NA | 4 | 2 | 0 | 0 |
| 43T | NA | NA | NA | NA | NA | 0 | 0 | 10 | NA | 9 | 2 | 0 | 0 |
| 51N | NA | NA | NA | NA | NA | 0 | 0 | 10 | NA | NA | 2 | 0 | 0 |
| 51T | NA | NA | NA | NA | NA | 0 | 0 | 10 | NA | 9 | 2 | 0 | 0 |
| 57N | NA | NA | NA | NA | N | NA | NA | 10 | NA | 2 | 2 | NA | NA |
| 57T | NA | NA | NA | NA | N | NA | NA | 10 | NA | NA | 2 | NA | NA |
| 59N | NA | NA | NA | NA | NA | 0 | 0 | 10 | NA | 28 | 2 | 0 | 0 |
| 59T | NA | NA | NA | NA | NA | 0 | 0 | 10 | NA | 47 | 2 | 0 | 0 |
| 64N | NA | NA | NA | NA | NA | 0 | 0 | 10 | NA | 8 | 2 | 0 | 0 |
| 64T | NA | NA | NA | NA | NA | 0 | 0 | 10 | NA | 10 | 2 | 0 | 0 |
| 31N | NA | NA | NA | NA | NA | 0 | 0 | 10 | NA | 0 | 2 | 0 | 0 |
| 31T | NA | NA | NA | NA | NA | 0 | 0 | 10 | NA | 4 | 2 | NA | 0 |
| 71T | MSI-H | IHC | NA | MSH6, MSH2 | Y | NA | NA | 36 | NA | 107 | 164 | NA | NA |
| 71N | MSI-H | IHC | NA |  | Y | NA | NA | 56 | NA | NA | 252 | NA | NA |
| 72T | MSI-H | IHC | NA | MSH2 | N | NA | NA | 13 | NA | 260 | 59 | NA | NA |
| 72N | MSI-H | IHC | NA |  | N | NA | NA | 35 | NA | 4091 | 156 | NA | NA |
| 73T | MSI-H | IHC | NA | MSH6 | N | NA | NA | 19 | NA | NA | 87 | NA | NA |
| 73N | MSI-H | IHC | NA |  | N | NA | NA | 18 | NA | NA | 79 | NA | NA |
| 74T | MSI-H | IHC | NA | MSH6 | NA | NA | NA | 3 | NA | NA | 14 | NA | NA |
| 74N | MSI-H | IHC | NA |  | NA | NA | NA | 9 | NA | NA | 39 | NA | NA |
| 75T | MSI-H | IHC | NA | MSH6 | NA | NA | NA | 86 | NA | 526 | 385 | NA | NA |
| 76T | MSI-H | IHC | NA | MLH1 | N | NA | NA | 42 | NA | 124 | 188 | NA | NA |
| 76N | MSI-H | IHC | NA |  | N | NA | NA | 61 | NA | NA | 276 | NA | NA |
| 77T | MSI-H | IHC | NA | MLH1 | NA | NA | NA | 77 | NA | NA | 346 | NA | NA |
| 78T | MSI-H | IHC | NA | MLH1 | NA | NA | NA | 5 | NA | 27291 | 24 | NA | NA |
| 79T | MSI-H | IHC | NA | MLH1 | NA | NA | NA | 55 | NA | 1888 | 248 | NA | NA |
| 80T | MSI-H | IHC | NA | MLH1 | NA | NA | NA | 457 | NA | 1587 | 2056 | NA | NA |
| 81T | MSI-H | IHC | NA | MLH1 | NA | NA | NA | 82 | NA | 221 | 367 | NA | NA |
| 82T | MSI-H | IHC | NA | MLH1, MSH2 | NA | NA | NA | 2968 | NA | NA | 13356 | NA | NA |
| 83T | MSI-H | IHC | NA | MSH2, MSH6 | NA | NA | NA | 242 | NA | NA | 1088 | NA | NA |
| 84T | MSI-H | IHC | NA | MSH2 | NA | NA | NA | 26 | NA | 262 | 117 | NA | NA |
| 85T | MSI-H | IHC | NA | MSH2 | Y | NA | NA | 43 | NA | 6642 | 194 | NA | NA |
| 85N | MSI-H | IHC | NA | NA | Y | NA | NA | 91 | NA | 22818 | 410 | NA | NA |
| 86T | MSI-H | IHC | NA | MSH2, MLH1 | NA | NA | NA | 74 | NA | NA | 331 | NA | NA |
| 87T | MSS | IHC | NA | NA | Y | NA | NA | 21 | NA | NA | 94 | NA | NA |
| 88T | MSI-H | IHC | NA | MLH1 | NA | NA | NA | 25 | NA | 342 | 112 | NA | NA |
