# Supplementary material for: Quantitative Profiling of Colorectal Cancer-Associated Bacteria Reveals Associations between Fusobacterium spp., Enterotoxigenic Bacteroides fragilis (ETBF) and Clinicopathological Features of Colorectal Cancer
Source: PLoS One. 2015 Mar 9;10(3):e0119462. doi: 10.1371/journal.pone.0119462 (PMC4353626; doi:10.1371/journal.pone.0119462)
Supplement: S2 Table — (DOCX) [file pone.0119462.s002.docx]

Table S2. Primers and their limits of detection (LODs) and qPCR efficiencies.

| **Target** | **Test gene** | **F & R primers (5'–3')** | **Product (bp)** | **Annealing T (C)** | **qPCR efficiency (%)** | **qPCR error** | **LOD*** | **% +ve at LOD** |
| --- | --- | --- | --- | --- | --- | --- | --- | --- |
| EPEC | *eaeA*^25^ | F-GTGACGATGGGGATCGAT |  |  |  |  |  |  |
|  |  | R1-ACGGCTGCCTGATAATGTT | 150 | 70–60 | 87 | 0.010 | 4 (20fg) | 73 |
|  | *eaeA* (intimin epsilon, gamma, zeta, alpha, pi, rho, beta, lambda, iota, kappa, eta, delta, xi, mu, kapp, jota) | R2-GGAACTGCATTGAGTAAAGGAG | 70 | 70-60 | 80 | 0.055 | 4 (20fg) | 100 |
|  | *eaeA* (intimin theta) | R3-GAAGCTGCATTGAGTAAAGAAG | 70 | 60 | ND | ND | ~ 10^#^ | ND |
|  | *bfp*^35^ | F-GGAAGTCAAATTCATGGGGGTAT |  |  |  |  |  |  |
|  |  | R-GGAATCAGACGCAGACTGGTAGT | 299 | 70–64 | 92 | 0.010 | 20 (100fg) | 100 |
| EHEC | *stx1*^36^ | F-ACATTGTCTGGTGACAGTAGC | 114 | 70–60 | 86 | 0.008 | 16 (100fg) | 83 |
|  |  | R-CGACATTAAATCCAGATAAGAAGTAGT |  |  |  |  |  |  |
|  | *stx2*^36^ | F-ATGACAACGGACAGCAGTTAT |  |  |  |  |  |  |
|  |  | R-CTGAACTCCATTAACGCCAGATA | 116 | 70–60 | 89 | 0.015 | 16 (100fg) | 100 |
| AIEC | *Clb*^28^ *(pks)* | F-GCGCATCCTCAAGAGTAAATA |  |  |  |  |  |  |
|  |  | R-GCGCTCTATGCTCATCAACC | 280 | 60 | 80 | 0.022 | 20 (100fg) | 50 |
|  | *afaC*^37^ | F-GCGCTATGTGGTGCAGAGTA |  |  |  |  |  |  |
|  |  | R-AAAACCGGTATTCACCAGGA | 185 | 65–60 | 86 | 0.026 | 20 (100fg) | 53 |
| *E. faecalis* | 16S rRNA^38^ | F-CCGAGTGCTTGCACTCAATTGG |  |  |  |  |  |  |
|  |  | R-CTCTTATGCCATGCGGCATAAAC | 137 | 60 | 80 | 0.017 | 12 (10 fg) | 87 |
| *S. gallolyticus* | *sodA*^9^ | F-CAATGACAATTCACCATGA |  |  |  |  |  |  |
|  |  | R-TTGGTGCTTTTCCTTGTG | 408 | 60–50 | 83 | 0.016 | 5 (20fg) | 100 |
| ETBF | *Bft1*^39^ | F-GACGGTGTATGTGATTTGTCTGAGAGA |  |  |  |  |  |  |
|  |  | R-ATCCCTAAGATTTTATTATCCCAAGTA | 294 | 65-55 | 78 | 0.020 | 4 (20fg) | 70 |
| *Fusobacterium* spp. | 16S rRNA^40^ | F-CGGGTGAGTAACGCGTAAAG |  |  |  |  |  |  |
|  |  | R1-GCCGTGTCTCAGTCCCCT | 228 | 60 | 85 | 0.026 | 9 (5fg) | 75 |
|  |  | R2-GCATTCGTTTCCAAATGTTGTCC | 61 | 60 | 83 | 0.020 | 9 (5fg) | 79 |
| FFPE QC | *cox1* | F-TATGGCGTTTCCCCGCATAA |  |  |  |  |  |  |
|  |  | R-GCGAGCAGGAGTAGGAGAGA | 69 | 57 | 98 |  | N/A | N/A |
