# Supplementary material for: Quantitative Profiling of Colorectal Cancer-Associated Bacteria Reveals Associations between Fusobacterium spp., Enterotoxigenic Bacteroides fragilis (ETBF) and Clinicopathological Features of Colorectal Cancer
Source: PLoS One. 2015 Mar 9;10(3):e0119462. doi: 10.1371/journal.pone.0119462 (PMC4353626; doi:10.1371/journal.pone.0119462)
Supplement: S3 Table — (DOCX) [file pone.0119462.s003.docx]

Table S3: qPCR conditions used.

| **Bacteria** | **Reagent** | **qPCR conditions** | **Number of cycles** |
| --- | --- | --- | --- |
|  |  |  |  |
| ETBF |  | 2 min @ 95°C |  |
|  | SensiFAST SYBR No Rox Mix (1x) | 1 s @ 95°C | x45 |
|  | F (200 nM final) | 5 s @ 65-55°C; decrease 0.5 °C/cycle |  |
|  | R (200 nM final) | acquire 1 s @ 80°C |  |
|  | template: 50 ng human DNA |  |  |
|  | H_2_0 |  |  |
|  | total = 20 μl |  |  |
|  |  |  |  |
|  |  |  |  |
| *E. faecalis* |  | 10 min @ 95°C |  |
|  | Maxima SYBR qPCR Master Mix (1x) | 15 s @ 95°C | x50 |
|  | F (900 nM final) | 60 s @ 60°C |  |
|  | R (900 nM final) | acquire @ 72°C |  |
|  | template: 50 ng human DNA |  |  |
|  | H_2_0 |  |  |
|  | total = 25 μl |  |  |
|  |  |  |  |
| *S. gallolyticus* |  | 2 min @ 95°C |  |
|  | SensiFAST SYBR No Rox Mix (1x) | 5 s @ 95°C | x60 |
|  | F (200 nM final) | 10 s @ 60-50°C, decrease 0.5 °C/cycle |  |
|  | R (200 nM final) | acquire @ 80°C |  |
|  | template: 50 ng human DNA |  |  |
|  | H_2_0 |  |  |
|  | total = 20 μl |  |  |
|  |  |  |  |
|  |  |  |  |
| EPEC (*bfpA*) |  | 2 min @ 95°C |  |
|  | SensiFAST SYBR No Rox Mix (1x) | 5 s @ 95°C | x60 |
|  | F (200 nM final) | 7 s @ 70-64°C; decrease 0.5 °C/cycle |  |
|  | R (200 nM final) | acquire @ 80°C |  |
|  | template: 50 ng human DNA |  |  |
|  | H_2_0 |  |  |
|  | total = 20 μl |  |  |
|  |  |  |  |
|  |  |  |  |
| EPEC (*eae*) | PCR buffer | 2 min @ 95°C |  |
|  | SensiFAST SYBR No Rox Mix (1x) | 5 s @ 95°C | x55 |
|  | F (200 nM final) | 7 s @ 70-60°C; decrease 0.5 °C/cycle |  |
|  | R (200 nM final) | acquire @ 80°C |  |
|  | template: 50 ng human DNA |  |  |
|  | H_2_0 |  |  |
|  | total = 20 μl |  |  |
|  |  |  |  |
| EHEC (*stx1*) |  | 2 min @ 95°C |  |
|  | SensiFAST SYBR No Rox Mix (1x) | 5 s @ 95°C | x60 |
|  | F (600 nM final) | 10 s @ 70-60°C; decrease 0.5 °C/cycle |  |
|  | R (600 nM final) | acquire @ 80°C |  |
|  | template: 50 ng human DNA |  |  |
|  | H_2_0 |  |  |
|  | total = 20 μl |  |  |
|  |  |  |  |
| EHEC (*stx2*) |  | 1 min @ 95°C |  |
|  | SensiFAST SYBR No Rox Mix (1x) | 1 s @ 95°C | x55 |
|  | F (400 nM final) | 5 s @ 70-60°C; decrease 0.5 °C/cycle |  |
|  | R (400 nM final) | acquire @ 80°C |  |
|  | template: 50 ng human DNA |  |  |
|  | H_2_0 |  |  |
|  | total = 20 μl |  |  |
|  |  |  |  |
| *Fusobacterium* spp. | | 10 min @ 95 |  |
|  | Maxima SYBR qPCR Master Mix (1x) | 15 s @ 95°C | x50 |
|  | F (300 nM final) | 45 s @ 60°C |  |
|  | R (300 nM final) | acquire @ 72°C |  |
|  | template: 50 ng human DNA |  |  |
|  | H_2_0 |  |  |
|  | total = 25 μl |  |  |
|  |  |  |  |
| AIEC (*afaC*) |  | 10 min @ 95 |  |
|  | Maxima SYBR qPCR Master Mix (1x) | 15 s @ 95°C | x50 |
|  | F (900 nM final) | 60 s @ 65-60 decrease 0.5 °C/cycle. |  |
|  | R (900 nM final) | acquire at 72°C |  |
|  | template: 50 ng human DNA |  |  |
|  | H_2_0 |  |  |
|  | total = 25 μl |  |  |
|  |  |  |  |
| AIEC (*ClB*) |  | 10 min @ 95 |  |
|  | Maxima SYBR qPCR Master Mix (1x) | 15 s @ 95°C | x60 |
|  | F (600 nM final) | 60 s @ 60°C |  |
|  | R (600 nM final) | acquire at 72°C |  |
|  | template: 50 ng human DNA |  |  |
|  | H_2_0 |  |  |
|  | total = 25 μl |  |  |
